# Tailoring the Host Range of *Ackermannviridae* Bacteriophages through Chimeric Tailspike Proteins

**DOI:** 10.3390/v15020286

**Published:** 2023-01-19

**Authors:** Jose Gil, John Paulson, Matthew Brown, Henriett Zahn, Minh M. Nguyen, Marcia Eisenberg, Stephen Erickson

**Affiliations:** 1 Laboratory Corporation of America Holdings, Los Angeles, CA 90062, USA; 2 Laboratory Corporation of America Holdings, New Brighton, MN 55112, USA; 3 Laboratory Corporation of America Holdings, Burlington, NC 27215, USA

**Keywords:** phage-based detection, bacteriophage, *Salmonella enterica*, luciferase reporter phage, *Ackermannviridae*, receptor-binding protein, tailspike protein

## Abstract

Host range is a major determinant in the industrial utility of a bacteriophage. A model host range permits broad recognition across serovars of a target bacterium while avoiding cross-reactivity with commensal microbiota. Searching for a naturally occurring bacteriophage with ideal host ranges is challenging, time-consuming, and restrictive. To address this, SPTD1.NL, a previously published luciferase reporter bacteriophage for *Salmonella*, was used to investigate manipulation of host range through receptor-binding protein engineering. Similar to related members of the *Ackermannviridae* bacteriophage family, SPTD1.NL possessed a receptor-binding protein gene cluster encoding four tailspike proteins, TSP1-4. Investigation of the native gene cluster through chimeric proteins identified TSP3 as the tailspike protein responsible for *Salmonella* detection. Further analysis of chimeric phages revealed that TSP2 contributed off-target *Citrobacter* recognition, whereas TSP1 and TSP4 were not essential for activity against any known host. To improve the host range of SPTD1.NL, TSP1 and TSP2 were sequentially replaced with chimeric receptor-binding proteins targeting *Salmonella*. This engineered construct, called RBP-SPTD1-3, was a superior diagnostic reporter, sensitively detecting additional *Salmonella* serovars while also demonstrating improved specificity. For industrial applications, bacteriophages of the *Ackermannviridae* family are thus uniquely versatile and may be engineered with multiple chimeric receptor-binding proteins to achieve a custom-tailored host range.

## 1. Introduction

Bacteriophages (phages) have significant potential to benefit multiple sectors of the commercial food industry. Phage-based biocontrol, bioremediation, and therapy have all been explored and previously discussed in-depth [1,2,3]. In addition to these applications, phages can also facilitate detection of foodborne bacterial pathogens, improving food safety and reducing illness [4]. Phage reporters are particularly effective when combined with other recent scientific advancements, such as the engineered and optimized luciferase NanoLuc^®^. NanoLuc^®^ is roughly one hundred times brighter than standard luciferases and has a small genetic footprint [5]. This intensity and size, combined with commercial availability, has renewed interest in luciferase reporter phages by offering unprecedented diagnostic sensitivity. NanoLuc^®^-encoding phages have now been successfully engineered to detect a variety of foodborne pathogens including *E. coli*, *Cronobacter*, *Salmonella*, and *Listeria* [6,7,8,9,10,11,12]. The continued development of this promising technology is of both scientific and commercial interest.

The performance of phage-based technologies is tied to their specificity, which is determined, in part, by host recognition through receptor-binding proteins (RBPs). RBPs are a diverse group of proteins capable of recognizing various surface structures, including lipopolysaccharides, wall teichoic acids, bacterial proteins, and bacterial capsules [13,14,15,16]. Tailspike proteins (TSPs) are a largely structurally conserved class of RBPs that commonly contain both receptor-binding and receptor-degrading activity [17]. TSPs form homotrimers, consisting of an N-terminal fragment mediating binding to the phage baseplate and a C-terminal fragment with receptor-binding and enzymatic activity [18]. These two fragments are typically connected by a small linker region, or neck, which likely provides structural flexibility and may also play a role in DNA ejection through signal transmission [19].

Determinants of host recognition are obvious targets for genetic manipulation in the pursuit of improved performance. A variety of strategies have been explored to modify phage specificity through engineering of TSPs and other RBPs [20,21]. One effective method employs the use of chimeras, and it takes advantage of the structural partitions between domains important for phage assembly and those important for receptor-binding. For example, a chimeric RBP of the *Listeria* phage PSA has been engineered with a native N-terminal fragment and foreign C-terminal fragment [22]. As expected, the host specificity of this chimeric phage was switched to that of the foreign C-terminus, whereas N-terminal interactions with the native base plate were preserved. A similar technique has also been used to change the host range of several *Klebsiella* phages [23]. The segmented structure of TSPs and other RBPs thus allows design of chimeric phages with modified host recognition without disrupting native assembly.

Engineering of phage host range is often limited to modifying the binding domain of a single RBP, replacing native host recognition in a one-to-one substitution. Phage encoding of multiple RBPs, however, may allow for more extensive and flexible customization, further improving performance [16,24,25,26]. In particular, members of the *Ackermannviridae* family of lytic phages (previously known as *Viunalikevirus*) utilize a branched RBP complex encoded by a gene cluster of up to four TSPs (TSP1-4) [27]. Critically, each TSP contains a C-terminal segment with independent and distinct specificity, often generating host ranges that span across genera. For example, CBA120, a well-described *Ackermannviridae* family member, is capable of infecting *Salmonella enterica* subsp. *Enterica* serovar Minnesota (*S.* Minnesota) through TSP1’s activity and *Escherichia coli* serovar O157, O77, and O78 through the activity of TSP2, TSP3, and TSP4, respectively [27,28]. Unsurprisingly, even with native host ranges, lytic phages of the *Ackermannviridae* family have shown promise for commercial applications within the food industry [29,30,31,32,33]. The *Ackermannviridae* family may thus represent a superior, yet largely unexplored, engineering platform for synthetic phages with custom-tailored host ranges.

*Salmonella* is a common cause of foodborne illness, a leading cause of hospitalization and death among foodborne pathogens and a major impediment to food safety in the United States [34]. Phage-based diagnostics have the potential to improve food safety if they can provide rapid, sensitive, and accurate detection of bacterial pathogens. Previously, an engineered NanoLuc^®^-encoding reporter phage, SPTD1.NL (formerly TSP1.NL), detected approximately 50% of tested *Salmonella* strains and cross-reacted with *Citrobacter sedlakii* [10]. SPTD1 (formerly TSP1) is a *Salmonella* phage isolated from sewage, and it is characterized by considerable DNA sequence homology to SFP10, a myovirus now in the *Ackermannviridae* family [10]. The purpose of this study was to improve the performance of SPTD1.NL through engineering of a TSP complex, common in *Ackermannviridae* family members. SPTD1 was found to encode for four TSPs, only one of which showed specificity for the target (*Salmonella*). Two *Salmonella*-specific chimeric TSPs were exchanged with noncontributory TSPs, ultimately leading to an improvement in reporter specificity and coverage. The results of this study highlight the potential of the *Ackermannviridae* family in generating synthetic phages with tailored host ranges. Synthetic phages with chimeric receptors may be particularly valuable in phage-based applications, as they can be easily modified to improve strain coverage or specificity. Furthermore, they may simplify phage cocktail development and limit the burdensome search for natural phages with ideal host ranges.

## 2. Materials and Methods

### 2.1. Bacterial Strains

Source and strain information for bacterial species utilized in this study is provided separately (Table S1). Where mentioned, *Salmonella* serogroup information was obtained from the WHO Collaborating Centre for Reference and Research on *Salmonella* [35]. Bacterial strains were routinely cultured at 37 °C in tryptic soy broth (TSB) (Oxoid, Hampshire, UK) with shaking at 225 revolutions per minute (RPM).

### 2.2. DNA, Amino Acid, and Structural In Silico Comparisons

The genomes of CBA120 (JN593240), Det7 (KP797973), and SPTD1 (OP991882) are publicly available. Sequence comparisons between the TSP clusters of CBA120 and SPTD1 were performed using EMBOSS Needle with default settings [36,37]. For sequence comparison, delineation of N- and C-terminal domains was based upon known crystal structures of CBA120’s TSP1-4 [27,38,39,40]. Whole genome comparisons and all other amino acid analyses were performed using BLAST^®^ with default settings [41]. Alignments of recipient, chimeric, and donor phages were performed using Clustal Omega and visualized with Jalview (version 2.11.2.5, University of Dundee, Dundee, Scotland) [42,43].

Computational predictions of the trimeric structure of TSP3 for CBA120, SPTD1, and a proposed chimeric TSP3 were generated using Alphafold2 [44]. Alphafold2 was run on Ubuntu Linux (version 22.04, Canonical Ltd., London, UK) in multimer mode with the reduced database (DBS) and otherwise default settings. Output files were then visualized within the SWISS-MODEL Workspace [45,46].

### 2.3. Phage Engineering

The isolation of the *Salmonella* phage SPTD1 and generation of the NanoLuc^®^-encoding recombinant, which is referred to as SPTD1.NL, formerly “TSP1” and “TSP1.NL”, have been described in detail previously [10]. The *E. coli* phage CBA120, obtained from Dr. Elizabeth Kutter, has also been characterized in prior work [28]. A NanoLuc^®^-encoding CBA120, referred to as CBA120.NL, was generated in a similar manner to SPTD1.NL, placing Nanoluc^®^ downstream of the major capsid protein, with the following major exceptions. To drive Nanoluc^®^ production in CBA120, a known *Ackermannviridae* late gene promoter was selected [25]. Additionally, an *E. coli* O157:H7 strain (43888) from the American Type Culture Collection (ATCC, Manassas, VA, USA) was used for the homologous recombination infection and subsequent recombinant purification.

RBP-CBA120-1 was designed to be CBA120.NL with a chimeric TSP3, encompassed by an N-terminal region (AA 1–157) from CBA120’s TSP3 and a C-terminal region from SPTD1’s TSP3 (AA 158–708) (Figure S1). The transition site between native and chimeric TSP3 was selected to be after Ala157, separating the N- and C-terminal functions by targeting a site a few amino acids into the “neck” or linker region according to published structures [27,39]. To achieve this, homologous recombination was facilitated by an upstream and downstream flank with homology to the phage surrounding the sequence to be exchanged. This approach has been described previously in detail [10]. For this design, the upstream flank consisted of 500 bp preceding the transition site, followed by the sequence encoding for the C-terminal region of SPTD1’s TSP3 (AA 158–708) and also the downstream flank consisting of 500 bp after the chimeric TSP. These sequences were ordered from Integrated DNA Technologies (IDT, Coralville, IA, USA) to be inserted into their plasmid, pUCIDT(Amp). The sequence of pUCIDT(Amp) is publicly available from IDT. Electroporation of these plasmids into host strains and subsequent phage infections were performed as generally described previously [10]. To generate RBP-CBA120-1 through homologous recombination, plasmid-carrying ATCC 43888 was infected with CBA120.NL. Compared to previous methods, a modified approach was used to isolate the desired recombinant. Following homologous recombination, lysates were diluted and plated using a standard double-layer agar method on ATCC 19585, a strain of *S.* Typhimurium. This strain was hypothesized to allow propagation of the desired recombinant but not the original CBA120.NL. As plaques were observed, they were picked and sequentially passaged at least three times for purity, as previously described [10]. This method of positive selection for chimeric TSPs allowed for rapid isolation and purification of recombinants.

RBP-CBA120-2 was designed similar to RBP-CBA120-1, with the following exceptions. The chimeric TSP2 consisted of an N-terminal region (AA 1–249) from CBA120’s TSP2 and a C-terminal region from SPTD1’s TSP2 (AA 250–729) (Figure S2). To achieve this, the sequence encoding for the C-terminal region of SPTD1’s TSP2 (AA 250–729) was used between the upstream flank and the downstream flank. Following homologous recombination, lysates were plated and plaques were purified on ATCC 51493, a strain of *C. sedlakii*.

RBP-SPTD1-1 was designed to be an SPTD1.NL with a chimeric TSP4, such that it would be encompassed by an N-terminal region (AA 1–479) from SPTD1’s TSP4 and a C-terminal region from CBA120’s TSP4 (AA 480–1036) (Figure S3). It was achieved similar to RBP-CBA120-1, with the following exceptions. After the upstream flank, the sequence encoding for the C-terminal region of CBA120’s TSP4 (AA 480–1036) was used. Homologous recombination was facilitated by infection of plasmid-containing ATCC 19585. Lysates were plated and plaques were purified on ECOR 70, an *E. coli* O78 strain.

RBP-SPTD1-2 was designed to be SPTD1.NL with a chimeric TSP1, encompassed by an N-terminal region (AA 1–149) from SPTD1’s TSP1 and a C-terminal region from CBA120’s TSP1 (AA 150–767) (Figure S4). It was achieved similarly to RBP-SPTD1-1, with the following exceptions. After the upstream flank, the sequence encoding for the C-terminal region of CBA120’s TSP1 (AA 153–770) was used. Lysates were plated and plaques were purified on 52329.1, a strain of *S.* Minnesota.

RBP-SPTD1-3 was designed to build upon RBP-SPTD1-2 with an additional chimeric TSP2 encompassed by an N-terminal region (AA 1–255) from SPTD1’s TSP2 and a C-terminal region from Det7’s TSP2 (AA 256–801) (Figure S5). It was achieved similarly to RBP-SPTD1-2, with the following exception. After the upstream flank, the sequence encoding for the C-terminal region of Det7’s TSP2 (AA 253–798) was used. Homologous recombination was facilitated via infection of plasmid-containing ATCC 19585 with RBP-SPTD1-2. Lysates were plated and plaques were purified on SLR 377, a strain of *S.* Anatum. A summary of both native and chimeric TSP clusters for all phages utilized in this study is provided (Table 1).

### 2.4. Phage Stock Preparation

Two types of phage preparation were used in this study: crude broth lysates and purified stocks. Broth lysates were used for only RBP-SPTD1-1, RBP-SPTD1-2, and RBP-CBA120-2, whereas purified stocks were prepared for all other phages. To generate these lysates, one isolated plaque of each phage was picked from a double-layer agar plate, resuspended into 4 mL of TSB, and combined with approximately 150 µL of log-phase bacterial culture. ECOR 70, 52329.1, and ATCC 51493 were used for RBP-SPTD1-1, RBP-SPTD1-2, and RBP-CBA120-2, respectively. This mixture was incubated at 37 °C with 225 RPM shaking until visual lysis was apparent. The lysate was then centrifuged for 10 min at 4700× *g,* and the supernatant was syringe filtered through a 0.45 µm Supor^®^ membrane (Pall Corp., Port Washington, NY, USA). Titer was determined by serial dilution and standard plaque counting methods.

Generation of high titer purified stocks of both wild-type and recombinant bacteriophages has been previously described in detail [10]. Briefly, broth lysates were first prepared via infection of log-phase bacterial cultures. ATCC 43888 was used for CBA120.NL, whereas ATCC 19585 was used for SPTD1.NL, RBP-CBA120-1, and RBP-SPTD1-3. Lysates were clarified and pelleted via ultracentrifugation. Pellets were resuspended in TMS (50 mM Tris-HCl pH 7.8, 10 mM MgCl_2_, and 300 mM NaCl), treated with DNAse I and RNAse, and further purified on a sucrose gradient (10–30%). Bands containing phage were pelleted and resuspended in TMS, and the titer was determined as described above.

### 2.5. Phage Spot Assay

Spot assays were conducted on a prepared double-layer agar as follows. Bacterial cultures in log phase were diluted to an optical density (OD_600_) of 0.2 in TSB. To achieve the desired density and layering, 300 µL of this dilution was combined with 3 mL of molten 0.5% TSB semi-solid and plated atop a TSB agar plate. Regardless of stock type (lysate or purified stock), phage preparations were diluted to approximately 1 × 10^8^ plaque forming units (PFU) per mL in TSB for spotting. Each spot consisted of 4 µL of phage dilution, which was pipetted onto the semi-solid layer in marked sections and allowed to incubate overnight at 37 °C. Results were recorded using a Gel Doc EZ Imager (Bio-Rad Laboratories, Hercules, CA, USA). A summary of activity associated with each TSP as observed from spot assays conducted with recombinant phages throughout this study is provided (Table 1).

### 2.6. Luciferase Reporter Phage Assays

Performance of RBP-SPTD1-3 compared to SPTD1.NL was evaluated using a modification of a previously described limit of detection assay [10]. Log-phase bacterial cultures of four strains, including one representative strain of *S.* Typhimurium, *S.* Minnesota, *S.* Anatum, and *C. sedlakii*, were diluted to achieve either 10, 100, 1000, or 10,000 colony forming units (CFU) per well. Wells with TSB only (no bacterial culture) were also prepared to assess background. At lower burdens (10 and 100 CFU), ten replicate wells were prepared for each strain, whereas for 1000, 10,000, and TSB-only conditions, six replicate wells were prepared for each strain. The sample volume was 100 µL per well. No enrichment period was used prior to a two-hour infection. Infection was initiated by adding 10 µL of a working stock of each phage preparation. For all luciferase reporter phage assays, only purified phage stocks were used to limit background signal. Working stocks were prepared by diluting purified phage stocks in SM buffer (50 mM Tris-HCl pH 7.5, 8 mM MgSO_4_·7H_2_O, 100 mM NaCl, and 0.01% (*w/v*) gelatin) to a titer of 1.2 × 10^7^ PFU per mL. After the two-hour infection, 65 µL of a luciferase detection solution was added to each well. This reagent was prepared as a mixture of 50 µL Nano-Glo^®^ buffer, 15 µL 5× Renilla lysis buffer, and 1 µL Nano-Glo^®^ substrate (Promega Corp., Madison, WI, USA). The signal was quantified as relative light units (RLU) by using a GloMax^®^ Navigator (Promega Corp., Madison, WI, USA). After addition of detection solution, wells were read twice following a 3 min wait time with a 1 s integration. These two back-to-back readings (technical replicates) were averaged to produce a single RLU value for each well.

In order to evaluate the specificity of luciferase reporters, a previously described protocol for exclusivity testing was used [10]. Briefly, overnight stationary phase cultures of nine strains, including one representative strain of *C. braakii*, *Serratia marcescens*, *Shigella flexneri*, and *S.* Typhimurium and five strains of *E. coli* from different serogroups, were diluted to an OD_600_ of 0.2. A sample of 100 µL was added to each well, expected to correspond to over 10 million CFU per well. Given this burden, only one well per condition was prepared. Samples of each strain were infected with the indicated reporter phage, and signal production was assessed as described above.

## 3. Results

### 3.1. The Genome of SPTD1 Encodes a TSP Gene Cluster

**Figure 1:**
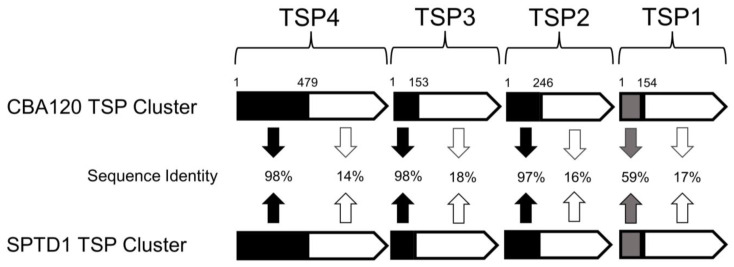
The genome of SPTD1 encodes for a TSP Cluster (ORF316, 318, 319, and 321) with significant N-terminal homology to the TSP Cluster of CBA120 (ORF 210–213). Comparisons of N- and C-terminal regions for each TSP were performed using EMBOSS Needle [36,37]. Protein amino acid sequence identity between CBA120 and SPTD1 for each TSP section is indicated, as are the boundaries of comparison. N-terminal regions of high (greater than 95%) and moderate (greater than 50%) sequence identity are indicated in black and grey, respectively. C-terminal regions with limited sequence identity (less than 20%) are indicated in white.

To confirm that SPTD1 utilized a branched RBP complex, which is typical of *Ackermannviridae* family members, the genomic sequence of SPTD1 was examined, annotated, and submitted to GenBank (OP991882). Whole genome BLAST^®^ analysis of SPTD1 revealed significant DNA homology (97% identity over 91% coverage) to CBA120 (JN593240), a prototypical member of the *Ackermannviridae* family with a well-described TSP complex [27,28,38,39,40,47]. Upon inspection, SPTD1 was found to possess homologs of each CBA120 TSP (Figure 1). Substantial N-terminal amino acid sequence similarity between CBA120 and SPTD1 TSPs, ranging from 59% identity for TSP1 to over 96% identity for TSP2, TSP3, and TSP4 was observed. The N-terminus of these TSPs serves an essential structural role, mediating both attachment to the baseplate (TSP4) and facilitating complex assembly through TSP interactions (TSP1-4) [27,40]. In contrast to the N-terminus, less than 20% amino acid identity was observed in the remaining sections of each protein. The C-terminus of each TSP is responsible for host specificity, as it possesses enzymatic and receptor-binding activity [27,38,39,47]. This dichotomy of conserved N-terminal structural TSP “heads” and divergent C-terminal receptor-binding TSP “bodies” within the *Ackermannviridae* family has been previously noted [48,49]. The contrast between the C-terminal receptor-binding domain of CBA120 and SPTD1’s TSPs correlates well with absence of overlapping hosts. CBA120 infects *E. coli* (O77, O78, and O157) and *S.* Minnesota, whereas SPTD1 has been reported to infect various non-Minnesota *Salmonella* serovars and *Citrobacter sedlakii* [10,27]. Thus, SPTD1 encodes for a RBP complex, and the host range of this phage is likely determined by the C-terminal section of each TSP within this gene cluster.

### 3.2. Sequence Comparison of SPTD1’s Tailspike Proteins to Known Ackermanniridae Phages

In CBA120, TSP1 is responsible for *S.* Minnesota (serogroup O:21) infection, TSP2 is responsible for *E. coli* O157 infection, TSP3 is responsible for *E. coli* O77 infection, and TSP4 is responsible for *E. coli* O78 infection [27]. In contrast, the contribution of each TSP in SPTD1 was not known. To determine if the receptor-binding regions of SPTD1 were conserved and had been previously described, BLAST^®^ analysis was performed on the amino acid sequence of each C-terminus against the *Ackermannviridae* family (taxid:2169529). For SPTD1’s TSP1 (155–615), top sequence alignment hits to the C-terminus were relatively weak (34% amino acid identity over 34% coverage). In contrast, the C-terminus of SPTD1’s TSP2 (252–729) was highly conserved (approximately 99% identity over 100% coverage) in six *Salmonella* phages (Chennai, vB_SenA_SM5, BRM 13314, Sh19, SE14, and vB-SalM-PM10). Although noteworthy, the specificity of this receptor-binding region has unfortunately not been determined in any of these phages. Thus, for TSP1 and TSP2, little information can be gathered by sequence analysis alone.

As with TSP2, the C-terminus of SPTD1’s TSP3 (154–708) was highly conserved in several related phages. Seven *Salmonella* phages (Mutine, vB-SalM-SJ3, vB_SalM-LPST94, vB-SalM-PM10, and ST-W77, barely, and Det7) and one *E. coli* phage (EP75) of the *Ackermannviridae* family shared approximately 99% identity over 100% coverage with the C-terminus of SPTD1’s TSP3. Importantly, prior investigations have revealed significant insight into the activity and specificity of this particular C-terminal sequence. In Det7 (KP797973), this sequence corresponds to a TSP3 homolog, which was found to bind to the O-antigen oligosaccharides of *S.* Typhimurium (serogroup O:4) [50]. In EP75 (MG748547), this sequence also corresponds to a TSP3 homolog, which was found to be enzymatically active against several *Salmonella* serovars, including Typhimurium and Derby (serogroup O:4); Enteritidis, Panama, and Javiana (serogroup O:9); and *S.* Braenderup (serogroup O:7) [49]. As previously noted for Det7, the C-terminus of SPTD1’s TSP3 has unexpected homology (61% amino acid identity over 99% coverage) to the C-terminus of the tailspike of P22, a podovirus of the *Lederbergvirus* genus [50]. Interestingly, the P22 tailspike protein recognizes *Salmonella* serogroups O:2, O:4, and O:9, and known catalytic residues (Asp-392, Asp-395, and Glu-359) appear conserved in SPTD1’s TSP3 [51]. Given that SPTD1 was previously shown to infect many serovars within these serogroups, it is likely that TSP3 significantly contributes to SPTD1’s recognition of *Salmonella* [10].

In respect to SPTD1’s TSP4, a close match to the C-terminus (480–1013) was also found in other *Ackermannviridae* phages. Nine *Salmonella* phages (moki, ST-W77, vB-SalM-SJ3, BRM 13312, BRM 13313, SenASZ3, SenALZ1, Se_AO1, and Sh19) and one *E. coli* phage (EP75) had greater than 95% identity over 100% coverage to this amino acid sequence. This receptor-binding region has been investigated previously, but its specificity ultimately remained elusive, as it failed to contribute to any portion of the known *Salmonella* and *E. coli* host range of EP75 [49]. Thus, like TSP1 and TSP2, the contributions of SPTD1’s TSP4 are not obvious from sequence alone.

### 3.3. Generation of Chimeric Ackermannviridae Tailspike Proteins

**Figure 2:**
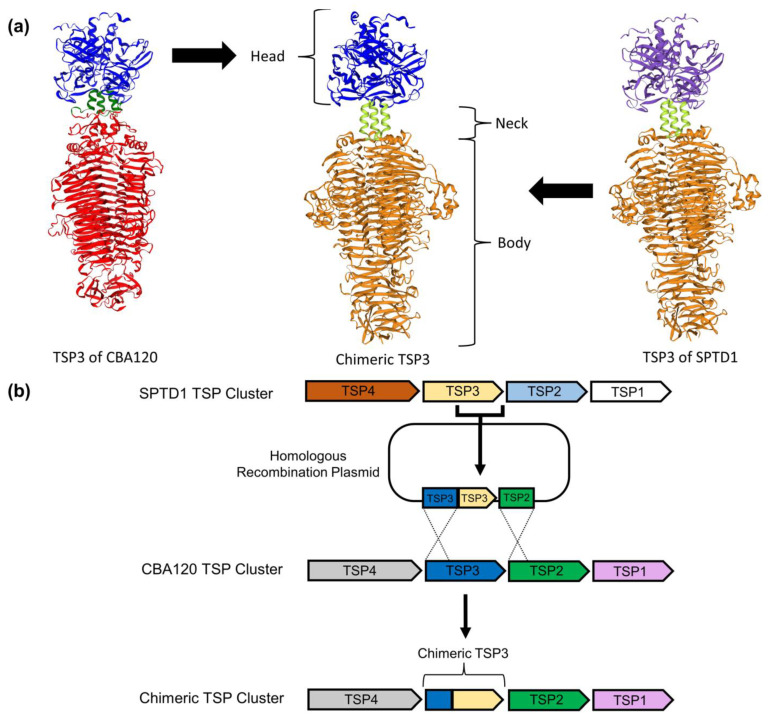
*Ackermannviridae* tailspike proteins can be engineered to generate chimeric receptor-binding proteins. Computational predictions (**a**) of the trimeric structure of TSP3 for CBA120, SPTD1, and the proposed chimeric TSP3 were generated using Alphafold2 and visualized with the SWISS-MODEL Workspace [44,45,46]. The predicted structure of CBA120’s TSP3 is highly similar to a previously reported crystal structure [39]. Color was used to visually distinguish regions and source for the proposed TSP3 chimera. Homologous recombination (**b**) was used to generate a chimeric TSP3 by replacing the C-terminus of CBA120’s TSP3 with the analogous region in SPTD1. Each TSP in the SPTD1 and CBA120 cluster was assigned a color at random to allow tracking throughout the engineering process.

Recombination between TSPs has been proposed to occur naturally downstream of the conserved N-terminus, providing a path to altered host range via swapping of C-terminal receptor binding domains [48]. To our knowledge, artificial engineering using this same approach has not been attempted within the *Ackermannviridae* family. Given the non-overlapping and distinct host ranges, CBA120 and SPTD1 were excellent candidates for exploring artificially generated chimeric TSPs. Additionally, the established specificity of each CBA120 TSP provides a solid foundation of expected phenotypes [27]. The conceptual approach to making a chimeric TSP involves the replacement of the C-terminal receptor-binding portion while maintaining the original N-terminal structural element. For example, a chimeric TSP3 could be designed with a recipient’s (CBA120’s) N-terminal domain and a donor’s (SPTD1’s) C-terminal domain (Figure 2a). In order to explore the viability of engineering chimeric TSPs, CBA120 was selected as the recipient for chimeras of TSP2 and TSP3 using SPTD1 as a donor, whereas SPTD1 was used as the recipient for chimeras of TSP1 and TSP4 with CBA120 as a donor. This strategy was expected to provide significant insight into the TSPs responsible for SPTD1’s host range as well as demonstrate the ability to engineer each member of the TSP complex. The selected chimeras were generated using a homologous recombination strategy (Figure 2b). NanoLuc^®^-encoding recombinants of CBA120 and SPTD1, which are referred to as CBA120.NL and SPTD1.NL, were used as recipients for all chimeras to facilitate downstream investigation of diagnostic utility. Positive selection using bacterial hosts specific to the donor allowed rapid isolation of chimeras following recombination. Using this strategy, phage preparations of the following chimeras were created, RBP-SPTD1-2 from SPTD1.NL with the TSP1 C-terminus of CBA120, RBP-CBA120-2 from CBA120.NL with the TSP2 C-terminus of SPTD1, RBP-CBA120-1 from CBA120.NL with the TSP3 C-terminus of SPTD1, and RBP-SPTD1-1 from SPTD1.NL with the TSP4 C-terminus of CBA120.

### 3.4. Functional Characterization of TSP Chimeras

**Figure 3:**
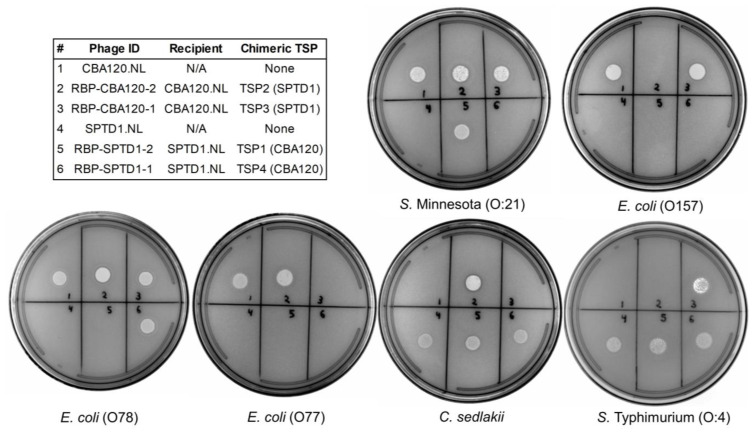
TSP chimeras are functional and allow customization of phage host range. Each phage preparation (1 × 10^8^ PFU/mL) was tested for specificity by spotting 4 µL onto bacterial lawns. The appearance of a circular transparent spot is evidence of phage activity against the bacterial strain. Agar plates were imaged using a Gel Doc EZ Imager (Bio-Rad Laboratories, Hercules, CA, USA). The legend indicates the identity of each spot number (#), including the recipient phage and donor TSP. Bacterial strain information is provided separately (Table S1).

To determine the specificity of the newly generated chimeric recombinants, a phage spot assay was used [52]. As expected, CBA120.NL spotted on *S.* Minnesota and *E. coli* serovars O157, O77, and O78, whereas SPTD1.NL spotted on *C. sedlakii* and *S.* Typhimurium (Figure 3). These results are in agreement with previously published native host ranges [10,27]. RBP-SPTD1-2 and RBP-SPTD1-1, which are recombinants of SPTD1 with chimeric TSPs from CBA120, produced unique spotting patterns. For example, RBP-SPTD1-2 gained the ability to spot on *S.* Minnesota, a phenotype associated with the donor TSP1 from CBA120. Similarly, RBP-SPTD1-1 gained the specificity of its TSP4 donor (CBA120), and it spotted on *E. coli* O78. Both of these SPTD1 chimeras maintained the ability to spot on *C. sedlakii* and *S.* Typhimurium, indicating that TSP1 and TSP4 of SPTD1 are not required for their recognition.

The chimeras of CBA120.NL, namely RBP-CBA120-2 and RBP-CBA120-1, also yielded novel spotting results (Figure 3). RBP-CBA120-1, which possessed a chimeric TSP3 from SPTD1, now spotted on *S.* Typhimurium instead of *E. coli* O77. This result matches prior predictions from sequence analysis and confirms that TSP3 from SPTD1 has activity against this serovar. Importantly, RBP-CBA120-1 maintained activity against *S.* Minnesota, *E. coli* O157, and *E. coli* O78, confirming that the chimeric TSP3 did not interfere with the functionality of the native TSP1, TSP2, and TSP4. RBP-CBA120-2, which possessed a chimeric TSP2 from SPTD1, now spotted on *C. sedlakii* instead of *E. coli* O157. As seen previously, activity of the non-chimeric TSPs was maintained with spotting on *S.* Minnesota, *E. coli* O77, and *E. coli* O78. This, once again, supports the lack of polar effects on non-engineered members of the tailspike complex. Finally, these data identify SPTD1’s TSP2 as the source of cross-reactivity against *Citrobacter*.

### 3.5. TSP3 Is Sufficient to Recapitulate SPTD1’s Known Salmonella Host Range

Although SPTD1’s TSP3 was confirmed to possess activity against *S.* Typhimurium, it was not yet known what portion of SPTD1’s *Salmonella* coverage this single TSP may be responsible for. To determine the extent of activity, a total of 22 additional *Salmonella* serovars from six serogroups known to be targeted by SPTD1 were investigated by spot assay. As expected, CBA120.NL had activity against zero of these serovars, whereas SPTD1.NL had activity against all 23 serovars (Table 2). Remarkably, RBP-CBA120-1 matched the activity of SPTD1 across all tested strains, whereas RBP-CBA120-2 had no activity against any *Salmonella* serovar. These data suggest that SPTD1’s TSP3 is sufficient for SPTD1’s recognition of *Salmonella* hosts.

### 3.6. Expansion and Contraction of Host Range through Chimeric Tailspike Proteins

As a diagnostic reporter, SPTD1.NL possessed several limitations, including cross-reactivity with *C. sedlakii* and incomplete coverage of *Salmonella* serovars [10]. Although coverage issues were largely resolved with the use of a phage cocktail, this adds complexity to reagent preparation and quality control. Furthermore, phage cocktails do not resolve detrimental cross-reactivity and often compound this issue. Given the solitary role of TSP3 in *Salmonella* specificity, it was of interest to determine if engineering non-contributory TSPs could be used to improve SPTD1.NL’s host range and eliminate cross-reactivity.

**Figure 4:**
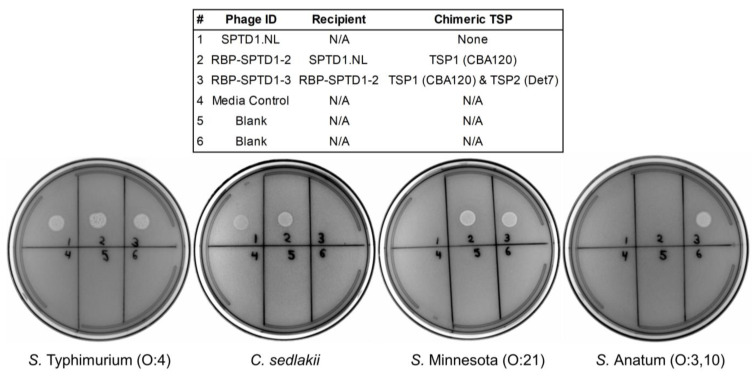
TSP chimeras of SPTD1.NL expand coverage of *Salmonella* and eliminate cross-reactivity with *Citrobacter*. Each phage preparation (1 × 10^8^ PFU/mL) was tested for specificity by spotting 4 µL on bacterial lawns. The appearance of a circular transparent spot is evidence of phage activity against the bacterial strain. Agar plates were imaged using a Gel Doc EZ Imager (Bio-Rad Laboratories, Hercules, CA, USA). The legend indicates the identity of each spot number including the original recipient phage and donor chimeric TSP. Bacterial strain information is provided separately (Table S1). The *S.* Anatum strain used was SLR 377. Media control indicates a spot without phages (TSB only), whereas blank is used to indicate an unused area.

Prior work in this study has already expanded the host range of SPTD1 to include *S.* Minnesota through a chimera of TSP1 from CBA120 (Figure 3). Because *S.* Minnesota is relevant for the food industry, particularly in Brazil (poultry), this recombinant (RBP-SPTD1-2) was used as a foundation for additional engineering [53]. In order to address cross-reactivity with *Citrobacter*, a chimeric TSP2 was pursued. The TSP2 from Det7 was a promising donor candidate, conveying specificity against an unrecognized *Salmonella* serovar, *S.* Anatum (serogroup O:3, 10) [48,54]. *S.* Anatum is also of significance to the food industry, and it was the source of an international outbreak due to contamination of infant formula [55]. A second round of engineering was thus performed to generate RBP-SPTD1-3, a double chimera of SPTD1.NL that contains a chimeric TSP1 (CBA120) and a chimeric TSP2 (Det7). Spot testing was used once again to reveal the activity of these recombinants (Figure 4). As expected, SPTD1.NL spotted only on *S.* Typhimurium and *C. sedlakii*, whereas RBP-SPTD1-2 had an expanded host range that also included *S.* Minnesota. The newly generated double-chimera (RBP-SPTD1-3) spotted on *S.* Typhimurium, *S.* Minnesota, and *S.* Anatum, correlating with the expected activity from the native TSP3, chimeric TSP1 (CBA120), and chimeric TSP2 (Det7), respectively. Importantly, the loss of the native TSP2 receptor-binding region was associated with the loss of spotting on *C. sedlakii*, which further supports the *Citrobacter* specificity of SPTD1’s TSP2. When summarized, these spot results suggest that RBP-SPTD1-3 has an optimized host range compared to SPTD1, which is defined by a broader recognition of *Salmonella* and reduced cross-reactivity.

### 3.7. RBP-SPTD1-3’s Activity against Other O:3-Containing Salmonella Serovars

The O:3, 10 *Salmonella* serogroup encompasses a large number of serovars with the potential for relevant diversity that may or may not impact recognition by RBP-SPTD1-3 [35]. Additionally, the *Salmonella* serogroup O:3, 10 is closely related to another serogroup, O:1, 3, 19. It has been suggested that these two groups should be combined due to their identical O-antigen gene clusters and remarkably similar structure [56,57]. Given this information, it was of interest to determine if RBP-SPTD1-3 had activity against serovars throughout both of these serogroups. For some serovars, multiple strains were assessed as available to validate the change in coverage between SPTD1.NL and RBP-SPTD1-3. A total of nine strains across three serovars were tested within the O:1, 3, 19 group, and a total of 21 strains across 11 serovars were tested within the O:3, 10 group. As expected, SPTD1.NL did not have native activity against any of these 30 strains (Table 3). RBP-SPTD1-3, however, gained activity against 14 of the 21 O:3, 10 group strains and 1 of the 9 O:1, 3, 19 group strains. This activity did not occur strictly by serovar. For example, *S.* Anatum, spotted on only three of five strains. Similarly, only one of two *S.* London strains and two of three *S.* Meleagridis strains yielded a positive spot assay result. Regardless of this variation, it is clear that overall RBP-SPTD1-3 had improved activity against the O:3, 10 serogroup of *Salmonella*, transitioning from 0% coverage to 67% coverage for these 21 strains. In contrast, RBP-SPTD1-3 yielded only minimal coverage of the O:1, 3, 19 group with activity against only one additional strain of the nine tested.

### 3.8. RBP-SPTD1-3 Is a Superior Luciferase Reporter for Salmonella Detection

**Figure 5:**
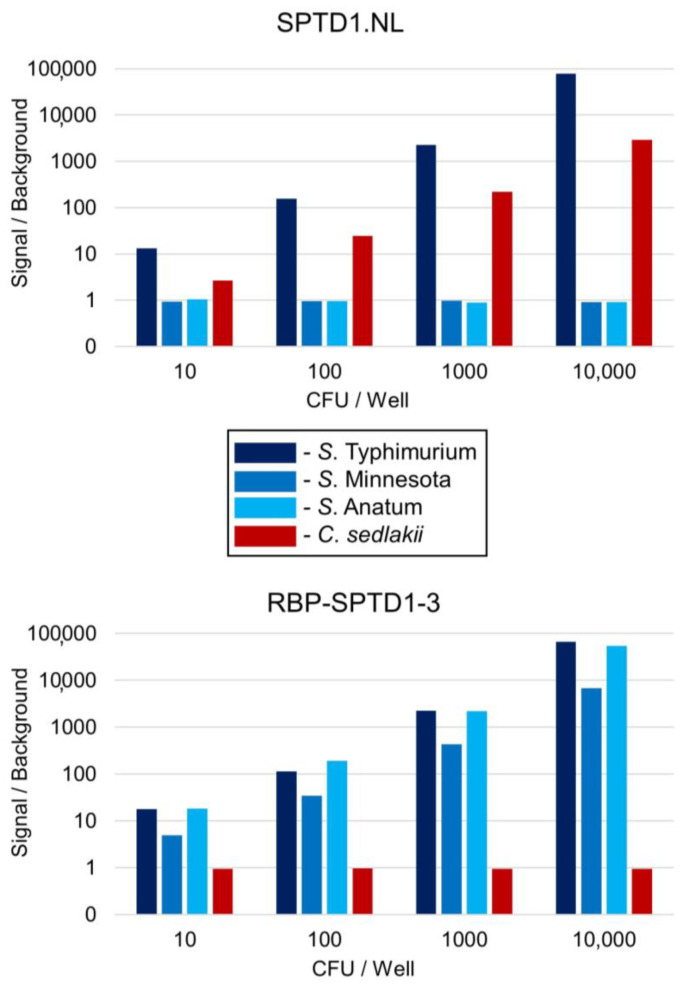
Compared to SPTD1.NL, RBP-SPTD1-3 has superior performance as a *Salmonella* diagnostic phage. Wells were prepared with log-phase cultures of each bacterial strain diluted to the indicated burden. Infection was initiated by adding 10 µL of a working stock (1.2 × 10^7^ PFU per mL) of SPTD1.NL (**Top**) or RBP-SPTD1-3 (**Bottom**) to each well. After two hours, Nanoluc^®^ production was assessed using a GloMax^®^ Navigator. Relative light units (RLU) values were obtained and averaged for replicate wells to determine signal. Background was calculated as the average RLU of replicate wells without bacteria (TSB only). As per the legend, *Salmonella* serovars are indicated by shades of blue, whereas *C. sedlakii* is in red. Bacterial strain information is provided separately (Table S1). The *S*. Anatum strain used was SLR 377. Individual RLU values for each well are provided separately (Table S2).

Given the improved spot assay results for RBP-SPTD1-3, it was of interest to determine if this chimeric recombinant was also a superior luciferase reporter when compared to its precursor, SPTD1.NL [10]. Bacterial burdens ranging from 10 colony forming units (CFU) to 10,000 CFU per well were examined without enrichment. Samples were infected with either SPTD1.NL or RBP-SPTD1-3 for two hours before NanoLuc^®^ production was quantified through light production. Signal was measured in relative light units (RLU) using a luminometer. As expected, SPTD1.NL generated a clear signal over background when the sample was *S.* Typhimurium or *C. sedlakii* (Figure 5). This signal could be detected as low as 10 CFU and increased in a burden-dependent manner up to 10,000 CFU per well. No signal was detected above background for *S*. Minnesota or *S*. Anatum. In contrast, RBP-SPTD1-3 generated a signal above background for all three *Salmonella* serovars. This signal was also burden-dependent from 10 to 10,000 CFU per well. Critically, RBP-SPTD1-3 no longer produced signal above background from *C. sedlakii* at any tested burden, confirming that the cross-reactivity with this species had been successfully eliminated. Raw RLU values for each strain and background are provided (Table S2). These results demonstrate that the chimeric TSPs engineered into RBP-SPTD1-3 contribute to both increased coverage and improved specificity, resulting in superior performance for *Salmonella* detection.

### 3.9. RBP-SPTD1-3 Does Not Exhibit Cross-Reactivity with Other Gram-Negative Bacteria

Previous work had identified SPTD1.NL as a *Salmonella*-specific reporter with the unique exception of cross-reactivity with *C. sedlakii* [10]. In contrast, the second *Salmonella* reporter phage previously engineered, SEA1.NL, possessed a substantially broader natural host range with cross-reactivity against several Gram-negative bacterial species, including strains of *E. coli*, *C. braakii*, *Serratia marcescens*, and *Shigella flexneri*. Given the changes made to RBP-SPTD1-3 to broaden the host range to additional *Salmonella* serogroups, it was important to confirm that this recombinant maintained *Salmonella* specificity. To evaluate exclusivity, a high burden (OD_600_ of 0.2) of several Gram-negative bacterial strains was infected with each reporter for two hours, and NanoLuc^®^ production was assessed. Despite the significant number of CFU, SPTD1.NL—as expected—yielded signal close to background for all samples except a positive control, *S.* Typhimurium (Table 4). The difference between background and positive control signal was substantial, roughly 100 RLU compared to over 800 million RLU. Importantly, RBP-SPTD1-3 demonstrated very similar performance and exhibited no evidence of cross-reactivity to any of these Gram-negative bacteria. Thus, the host range of RBP-SPTD1-3 has been expanded to include additional *Salmonella* strains without compromising *Salmonella* specificity.

## 4. Discussion

To the best of our knowledge, the branched RBP complex of the *Ackermannviridae* phage family had not previously been explored from an engineering perspective. The availability of four TSPs, each with their own distinct receptor-binding region, was hypothesized to allow significant customization of phage specificity (Figure 1). In the present study, chimeric TSPs were generated using homologous recombination and, thus, maintained native N-terminal regions important for structural assembly while substituting C-terminal receptor-binding regions (Figure 2). This approach proved successful with the *Ackermannviridae* RBP complex, just as it had with other phage families [22,23]. Chimeras of TSP1, TSP2, TSP3, and TSP4 were generated and conveyed the expected alternative specificity (Figure 3) (Table 1). Thus, each TSP of this cluster can be independently manipulated, regardless of their unique involvement in complex assembly and baseplate attachment [27,40]. Additionally, native TSPs were unaffected by engineering, as they preserved their specificity and confirmed the lack of polar effects on the complex as a whole (Figure 3). Overall, these data highlight the potential of the *Ackermannviridae* family as a flexible platform to build synthetic phages with customized host ranges.

Sequence analysis revealed nearly identical C-terminal binding regions for SPTD1’s, Det7’s, and EP75’s TSP3 and moderate similarity with the analogous receptor-binding region of P22. As anticipated based on these data, the specificity of SPTD1’s TSP3, as revealed by RBP-CBA120-1, closely aligned to previous results for Det7, EP75, and P22 (Table 2) [49,50,51]. Importantly, this work identified the TSP3 of SPTD1 as being sufficient for recognition of all 23 tested *Salmonella* serovars. In regards to the development of SPTD1 as a *Salmonella* reporter, this result suggested that SPTD1’s TSP1, TSP2, and TSP4 could be replaced without the loss of established *Salmonella* coverage and sensitivity [10].

*Citrobacter,* a close genetic relative of *Salmonella,* is ubiquitous in many food-related environments and has been previously noted as a source of false positives impeding accurate *Salmonella* detection [58,59]. When used as a luciferase reporter for *Salmonella* detection, signal production from SPTD1.NL appeared specific for *Salmonella* with the sole exception of *C. sedlakii* [10]. RBP-CBA120-2, that is, CBA120.NL with a chimeric TSP2 (SPTD1), demonstrated that this specificity is mediated by SPTD1’s TSP2 (Figure 3). This was further supported by the activity of RBP-SPTD1-3, which lost *C. sedlakii* spotting upon substitution of the native TSP2 (Figure 4). Despite the similarity between *Citrobacter* and *Salmonella*, these findings indicate that, at least for SPTD1’s recognition, the detection of these two species is distinct and can be separated. The exact nature of TSP2’s recognition of *C. sedlakii* remains unknown, although, like with many TSPs, species-specific O-antigen is a likely receptor candidate [60]. Importantly, this TSP2 receptor-binding region may help elucidate the host range of other phages as well. This region was highly conserved (99% identity) with six other *Salmonella* phages of the *Ackermannviridae* family (Chennai, vB_SenA_SM5, BRM 13314, Sh19, SE14, and vB-SalM-PM10). Of interest, many of these impacted *Salmonella* phages (vB_SenA_SM5, Sh19. SE14, and vB-SalM-PM10) are proposed to have commercial utility in food-related industries, although none have been formally evaluated for cross-reactivity against *C. sedlakii* to our knowledge [61,62,63,64]. The procedures demonstrated in this study may thus have value in improving the specificity of other phages currently in development.

The known host range of SPTD1 is conferred by TSP2 (*C. sedlakii*) and TSP3 (*Salmonella* serovars) (Figure 3) (Table 2). Given this finding, the activity of SPTD1’s TSP1 and TSP4 was uncertain. The C-terminal receptor-binding region of SPTD1’s TSP1 appears unique with minimal sequence similarity to other *Ackermannviridae* phages by BLAST^®^. On the other hand, the receptor-binding region of SPTD1’s TSP4 has significant homology to regions within several other phages, including EP75’s TSP4. Prior investigation of EP75 also failed to identify the specificity of this TSP4 sequence, as no activity against any known *Salmonella* or *E. coli* was observed [49]. It is noteworthy that SPTD1.NL was previously tested with a relatively large exclusivity panel, including 14 Gram-positive and 26 Gram-negative bacterial species, and no cross-reactivity outside of *C. sedlakii* was observed [10]. It is plausible that SPTD1’s TSP1 and TSP4 recognize a commensal species not included in this panel, as supplementary non-pathogenic hosts are likely to promote environmental persistence [65]. Another formal possibility is that the C-terminal regions of these two TSPs are nonfunctional. Because the N-terminal portions of TSP1 and TSP4 are important for complex assembly and are well-conserved, a functional C-terminal region is not expected to be essential (Figure 1) [40]. This prediction is supported by the existence of *Ackermannviridae* family members with a TSP complex consisting of only a single fully functional TSP. For example, the *Dickeya* phage LIMEStone1 has a TSP complex constructed from the N-terminal regions of two truncated TSPs (no receptor-binding domain) and one full-length TSP with a typical C-terminal domain [27]. Given these results and possibilities, further research will be required to determine the role of these TSPs.

As a luciferase reporter, SPTD1.NL provided coverage of approximately half of tested *Salmonella* serovars and cross-reacted with a single non-*Salmonella* species, *C. sedlakii* [10]. In the present study, genetic engineering of a chimeric TSP1 and TSP2 resulted in RBP-SPTD1-3, an improved recombinant with expanded activity against more *Salmonella* serovars and enhanced specificity (Figure 4) (Table 3). Critically, RBP-SPTD1-3 was also found to be an improved diagnostic tool for accurate detection of *Salmonella* (Figure 5). RBP-SPTD1-3 matched the capacity of SPTD1.NL for *S.* Typhimurium detection but also uniquely recognized *S.* Minnesota and *S.* Anatum. The detection of all three *Salmonella* serovars was evident at all burdens tested, including 10 CFU per well. These values over background were generated without bacterial enrichment and were immediately available following a two-hour infection and brief detection step. In addition to broader coverage of *Salmonella*, RBP-SPTD1-3 had improved specificity and yielded no signal over background for any tested burden, up to 10,000 CFU per well, of *C. sedlakii*. These improvements were not related to any unexpected changes to specificity, as cross-reactivity remained absent from other Gram-negative bacterial species, including *E. coli* serovars (O6, O79, O111, O121, and O145), *C. braakii*, *Serratia marcescens*, and *Shigella flexneri* (Table 4). From these data, it is clear that engineering the TSPs of *Ackermannviridae* phages led to a tangible improvement in utility as a diagnostic reporter. Importantly, the benefits of this approach are not likely to be limited to phage-based diagnostics within the food industry. Other applications, such as phage-based therapeutics, are also likely to benefit from improved accuracy through tailored host ranges.

RBP-SPTD1-3 gained activity on 67% of tested *Salmonella* strains within the O:3, 10 serogroup (Table 3). The activity on O:3, 10 strains was not directly connected to particular serovars. The chimeric TSP2 of RBP-SPTD1-3 is derived from the C-terminus of TSP2 from Det7, which was determined to have activity against *S.* Anatum via the O-antigen [54]. The receptor-binding region of this TSP was also recognized in this previous study to have homology to the *Salmonella* phage epsilon 15. Furthermore, both TSPs were described as endorhamnosidases, targeting the α-1,3-glycosidic bond between rhamnose and galactose in the LPS of *S.* Anatum [54,66]. Importantly, members of the O:3, 10 serogroup can possess three different O-antigens by serology, either O:3, 10, O:3, 15, and O:3, 15, 34, independently of serovar [35]. Critically, lysogenization of an O:3, 10 strain by two phages, epsilon 15 and 34, leads to changes in the O-antigen and seroconversion [67]. For example, an O:3, 10 strain lysogenized by epsilon 15 will become an O:3, 15 strain, and this strain can then be lysogenized by epsilon 34 to become an O:3, 15, 34 strain [56]. Lysogens of epsilon 15, such as O:3,15 strains, are resistant to superinfection due to the inability of epsilon 15 to bind to the modified O-antigen [66,68]. Given this, it is plausible that the partial recognition of strains within this serogroup is due to the activity of these temperate seroconverting phages. The mechanism behind RBP-SPTD1-3’s limited activity on strains in the O:1, 3, 19 group was unknown, but similar modifications to the conserved O-antigen structure could also restrict phage adsorption in this case [56]. Alternatively, a variety of phage resistance mechanisms have been described that could act downstream of phage adsorption and interfere with RBP-SPTD1-3 [69]. Future studies may benefit from investigating these phenotypes outside of phage infection by confirming the expressed O-antigen structure of each strain and directly monitoring sensitivity to the enzymatic activity of purified TSPs.

TSP4 mediates the critical attachment of the entire TSP complex to the phage base plate [27,40]. Despite this unique role, a chimeric TSP4 was successfully generated (RBP-SPTD1-1), confirming the availability of this site for engineering (Figure 3). Further improvement to RBP-SPTD1-3 may thus be feasible via a third chimeric TSP at this location. This may be beneficial, not only to add additional *Salmonella* coverage, but also to eliminate the potential for unexpected activity from the native TSP4 (unknown specificity). This would not be unprecedented as, for example, it took roughly eight years for the cross-genus activity of the *E. coli* phage CBA120, initially described as specific for *E. coli* O157, to be discovered on *S. enterica* [27,28]. Despite the feasibility of an additional chimeric TSP to RBP-SPTD1-3, there is a lack of *Ackermannviridae* TSPs capable of further supplementing *Salmonella* coverage at this time. For example, a recent study performed a comprehensive genetic analysis of TSPs from 99 *Ackermannviridae* phages [48]. In this in silico study, subtypes of TSPs were found that convey specificity against O:3, 10, O:4, O:9, and O:21 *Salmonella* serogroups. Unfortunately, RBP-SPTD1-3 already has activity for serogroup O:4 and O:9 from the native TSP3, for serogroup O:21 from the chimeric TSP1 (CBA120), and for serogroup O:3, 10 from the chimeric TSP2 (Det7) (Table 1). Thus, although TSP4 is available for further manipulation, a TSP donor to further expand coverage to additional *Salmonella* serogroups was not apparent. Candidates are likely to emerge from further discovery and characterization of *Ackermannviridae* phages, representing a constantly growing repertoire of TSPs available for this engineering method. Future studies may also benefit from expanding beyond *Ackermannviridae* TSPs and, for example, attempting to synthetically replicate the transfer of C-terminal receptor-binding domains from other phage families that is thought to be the evolutionary origin of some *Ackermannviridae* TSPs [50].

Overall, this work has led to three primary conclusions. First, the *Ackermannviridae* RBP complex has significant plasticity and can support synthetic host ranges through receptor-binding domain engineering of any TSP. Second, SPTD1’s TSP2 and TSP3 have activity against *C. sedlakii* and *Salmonella*, respectively, and these two TSPs are sufficient for the known host range of this phage. Third, improved diagnostic reporters with augmented coverage and specificity, such as RBP-SPTD1-3, can be generated by engineering multiple sites within the *Ackermannviridae* RBP complex. Given this, the *Ackermannviridae* family has significant potential to improve the performance of phage-based applications when the members of this family are used as synthetic phages with tailored host ranges. Specific improvements that may practically benefit existing applications include the elimination of false positives due to cross-reactivity, broader coverage of diversity within of a target bacterial species, and a reduced reliance on finding and maintaining natural phage cocktails. The limits of phage engineering are ultimately unknown, and future studies may unlock further improvements to this technology through the generation of synthetic phage of increased complexity and diversity.

## Figures and Tables

**Table 1:** Summary of Phage Design and Observed TSP Activity.

	Origin of C-Terminal Region of Each TSP ^1^				Bacterial Species and Serogroups Targeted by Each TSP ^2^			
Phage ID	TSP1	TSP2	TSP3	TSP4	TSP1	TSP2 ^3^	TSP3 ^4^	TSP4
CBA120.NL	CBA120_Native_	CBA120_Native_	CBA120_Native_	CBA120_Native_	*S. enterica* O:21	*E. coli* O157	*E. coli* O77	*E. coli* O78
SPTD1.NL	SPTD1_Native_	SPTD1_Native_	SPTD1_Native_	SPTD1_Native_	-	*C. sedlakii*	*S. enterica* O:4, O:9	-
RBP-CBA120-1	CBA120_Native_	CBA120_Native_	SPTD1_Chimera_	CBA120_Native_	*S. enterica* O:21	*E. coli* O157	*S. enterica* O:4, O:9	*E. coli* O78
RBP-CBA120-2	CBA120_Native_	SPTD1_Chimera_	CBA120_Native_	CBA120_Native_	*S. enterica* O:21	*C. sedlakii*	*E. coli* O77	*E. coli* O78
RBP-SPTD1-1	SPTD1_Native_	SPTD1_Native_	SPTD1_Native_	CBA120_Chimera_	-	*C. sedlakii*	*S. enterica* O:4, O:9	*E. coli* O78
RBP-SPTD1-2	CBA120_Chimera_	SPTD1_Native_	SPTD1_Native_	SPTD1_Native_	*S. enterica* O:21	*C. sedlakii*	*S. enterica* O:4, O:9	-
RBP-SPTD1-3	CBA120_Chimera_	Det7_Chimera_	SPTD1_Native_	SPTD1_Native_	*S. enterica* O:21	*S. enterica* O:3, 10	*S. enterica* O:4, O:9	-

^1^ All tailspike proteins (TSPs) have a native N-terminal sequence and either a native or chimeric receptor-binding C-terminal region, as indicated. ^2^ Primary activity associated with each TSP as observed from spot assays conducted on recombinant phages throughout this study. A hyphen is used to indicate TSPs in which activity was not observed. ^3^ Phages containing TSP2 Det7_Chimera_ of RBP-SPTD1-3 also demonstrated activity on one of nine *Salmonella* strains of serogroup O:1,3,19. ^4^ Phages containing the TSP3 SPTD1_Native_ to SPTD1.NL also demonstrated activity on a single *Salmonella* strain of the O:2, O:7, O:8, and O:35 serogroups.

**Table 2:** Activity of CBA120.NL with Native or Chimeric TSPs on *Salmonella* Serovars Targeted by SPTD1.NL.

		Spot Assay Result ^2^			
Serogroup	*S*. Serovar ^1^	CBA120.NL	RBP-CBA120-2	RBP-CBA120-1	SPTD1.NL
O:2	Paratyphi A	Negative	Negative	Positive	Positive
O:4	Abony	Negative	Negative	Positive	Positive
	Agona	Negative	Negative	Positive	Positive
	Bispebjerg	Negative	Negative	Positive	Positive
	Brandenburg	Negative	Negative	Positive	Positive
	Chester	Negative	Negative	Positive	Positive
	Derby	Negative	Negative	Positive	Positive
	Heidelberg	Negative	Negative	Positive	Positive
	Kiambu	Negative	Negative	Positive	Positive
	Paratyphi B	Negative	Negative	Positive	Positive
	Saintpaul	Negative	Negative	Positive	Positive
	Sandiego	Negative	Negative	Positive	Positive
	Schwarzengrund	Negative	Negative	Positive	Positive
	Typhimurium ^3^	Negative	Negative	Positive	Positive
O:7	Paratyphi C	Negative	Negative	Positive	Positive
O:8	Newport	Negative	Negative	Positive	Positive
O:9	Dublin	Negative	Negative	Positive	Positive
	Enteritidis	Negative	Negative	Positive	Positive
	Gallinarum	Negative	Negative	Positive	Positive
	Javina	Negative	Negative	Positive	Positive
	Panama	Negative	Negative	Positive	Positive
	Typhi	Negative	Negative	Positive	Positive
O:35	Alachua	Negative	Negative	Positive	Positive

^1^ Strain information is provided separately (Table S1). ^2^ A positive result indicates the visual appearance of a spot when 4 µL of the phage preparation (1 × 10^8^ PFU/mL) was placed upon a lawn of each indicated *S. enterica* subsp. *enterica* serovar. ^3^ Spot assay results for *S.* Typhimurium were shown previously (Figure 3) and are listed above for comparison.

**Table 3:** Comparison of SPTD1.NL and RBP-SPTD1-3 Activity on O:3-Containing *Salmonella* Serovars.

			Spot Assay Result ^2^	
Serogroup	*S*. Serovar ^1^	Strain ID	SPTD1.NL	RBP-SPTD1-3
O:1, 3, 19	Liverpool	AUG365	Negative	Negative
	Senftenberg	12004	Negative	Negative
		31072.1	Negative	Negative
		43845	Negative	Negative
		15106q	Negative	Negative
		SARB59	Negative	Negative
		SEP160	Negative	Positive
		SL1315	Negative	Negative
	Taksony	32133	Negative	Negative
O:3, 10	Amsterdam	41084	Negative	Positive
	Anatum ^3^	31064.1	Negative	Positive
		DMSO13	Negative	Negative
		NOV091	Negative	Negative
		SARB2	Negative	Positive
		SLR 377	Negative	Positive
	Benfica	AUG071	Negative	Positive
	Give	9268	Negative	Positive
		63213	Negative	Positive
	Lexington	11646	Negative	Negative
		9492-M	Negative	Negative
	London	43290	Negative	Negative
		JUL218	Negative	Positive
	Meleagridis	92	Negative	Negative
		11008.1	Negative	Positive
		FEB095	Negative	Positive
	Muenster	31053	Negative	Positive
		OCT084	Negative	Positive
	Uganda	51278.2	Negative	Positive
	Wagadugu	53298	Negative	Negative
	Weltevreden	BAA-2568	Negative	Positive

^1^ Additional strain information is provided separately (Table S1). ^2^ A positive result indicates the visual appearance of a spot when 4 µL of the phage preparation (1 × 10^8^ PFU/mL) was placed upon a lawn of each indicated *S. enterica* subsp. *enterica* serovar. ^3^ Spot assay results for the *S.* Anatum strain SLR 377 were shown previously (Figure 3) and are reproduced above.

**Table 4:** Comparison of SPTD1.NL and RBP-SPTD1-3 Reporter Specificity with High Bacterial Burdens.

		Relative Light Units ^3^	
Sample ^1^	Serogroup ^2^	SPTD1.NL	RBP-SPTD1-3
*Citrobacter braakii*	-	145	206
*Escherichia coli*	O6	94	152
	O79	150	178
	O111	242	255
	O121	141	190
	O145	165	209
*Serratia marcescens*	-	105	213
*Shigella flexneri*	-	102	204
*Salmonella* Typhimurium	O:4	801,150,016	1,007,500,000
Media Control	-	96	156

^1^ Overnight stationary phase cultures were diluted to an OD_600_ of 0.2 and infected for two hours with 10 µL of the indicated reporter phage working stock (1.2 × 10^7^ PFU per mL). Media controls without bacteria were included to evaluate background signal from media, each phage reporter, and detection reagents alone. ^2^ Strain information is provided separately (Table S1). ^3^ Following infection, Nanoluc^®^ production was assessed with a GloMax^®^ Navigator. Relative light units (RLUs) were averaged from two back-to-back reads.

## Data Availability

Data are contained within the article or supplementary material with the exception of the genome sequence and annotation of SPTD1 (previously TSP1). This information has been submitted to GenBank (OP991882) to be released with the publication of this manuscript. Availability of the engineered bacteriophages described in this study and those generated previously may require a material transfer agreement covering potential commercial applications.

## Supplementary Materials

The following supporting information can be downloaded at: https://www.mdpi.com/article/10.3390/v15020286/s1, Table S1: List of Bacterial Strains Used in This Study. Table S2: Individual Relative Light Unit Values for (Figure 5). Figure S1: Alignment of TSP3 from CBA120.NL, SPTD1.NL, and RBP-CBA120-1. Figure S2: Alignment of TSP2 from CBA120.NL, SPTD1.NL, and RBP-CBA120-2. Figure S3: Alignment of TSP4 from CBA120.NL, SPTD1.NL, and RBP-SPTD1-1. Figure S4: Alignment of TSP1 from CBA120.NL, SPTD1.NL, and RBP-SPTD1-2. Figure S5: Alignment of TSP2 from Det7, RBP-SPTD1-2, and RBP-SPTD1-3. References [35,36,42,43] are cited in the supplementary materials.
