# Supplementary material for: Tailoring the Host Range of Ackermannviridae Bacteriophages through Chimeric Tailspike Proteins
Source: Viruses. 2023 Jan 19;15(2):286. doi: 10.3390/v15020286 (PMC9965104; doi:10.3390/v15020286)
Supplement: Supplementary file 1 [file viruses-15-00286-s001.zip › viruses-2142404-supplementary.pdf]

Tailoring the Host Range of *Ackermannviridae* Bacteriophages through Chimeric Tailspike Proteins

Jose Gil [1], John Paulson [2], Matthew Brown [3], Henriett Zahn [2], Minh M. Nguyen [2], Marcia Eisenberg [3], and Stephen Erickson [2,*]

[1] Laboratory Corporation of America Holdings, Los Angeles, CA 90062, USA; gilj@labcorp.com
[2] Laboratory Corporation of America Holdings, New Brighton, MN 55112, USA; paulsoj@labcorp.com (J.P.); zahnh@labcorp.com (H.Z.); nguyem5@labcorp.com (M.M.N.)
[3] Laboratory Corporation of America Holdings, Burlington, NC 27215, USA; browm49@labcorp.com (M.B.); eisenbm@labcorp.com (M.E.)
* Correspondence: erickss@labcorp.com

Table S1. List of Bacterial Strains Used in This Study

Bacteria	Serogroup[1]	Serovar	Strain ID	Source[2]
Citrobacter braakii	-	-	51113	ATCC
Citrobacter sedlakii	-	-	51493	ATCC
Escherichia coli	O6	-	Nissle	USDA
	O77	-	23537	ATCC
	O78	-	ECOR70	MSU
	O79	-	ECOR5	MSU
	O111	-	33780	ATCC
	O121	-	BAA-2190	ATCC
	O145	-	BAA-2216	ATCC
	O157	-	43888	ATCC
Salmonella enterica	O:1,3,19	Liverpool	AUG365	USDA
		Senftenberg	12004	ATCC
			31072.1	USDA
			43845	ATCC
			15106q	UGA
			SARB59	FDA
			SEP160	USDA
			SL1315	FDA
		Taksony	32133	USDA
	O:2	Paratyphi A	9150	ATCC
	O:3,10	Amsterdam	41084	USDA
		Anatum	31064.1	USDA
			DMSO13	UIA
			NOV091	USDA
			SARB2	FDA
			SLR 377	UGA
		Benfica	AUG071	USDA
		Give	9268	ATCC
			63213	USDA
		Lexington	11646	ATCC
			9492-M	UGA
		London	43290	USDA
			JUL218	USDA
		Meleagridis	92	UGA
			11008.1	USDA
			FEB095	USDA
		Muenster	31053	USDA
			OCT084	USDA
		Uganda	51278.2	USDA
		Wagadugu	53298	USDA
		Weltevreden	BAA-2568	ATCC
	O:4	Abony	BAA-2162	ATCC
		Agona	SARB1	FDA
		Bispebjerg	9842	ATCC
		Brandenburg	AUG053	USDA
		Chester	11997	ATCC
		Derby	SARB11	FDA
		Heidelberg	SL476	FDA
		Kiambu	51316	USDA
		Paratyphi B	10719	ATCC

**Bacteria**	**Serogroup**[1]	**Serovar**	**Strain ID**	**Source**[2]
		Saintpaul	9712	ATCC
		Sandiego	APR025	USDA
		Schwarzengrund	13092.2	USDA
		Typhimurium	19585	ATCC
	O:7	Paratyphi C	BAA-1714	ATCC
	O:8	Newport	MH57137	UGA
	O:9	Dublin	SARB13	FDA
		Enteritidis	SL1302	FDA
		Gallinarum	4-50-39	UIA
		Javina	10721	ATCC
		Panama	SARB39	FDA
		Typhi	6539	ATCC
	O:21	Minnesota	52329.1	USDA
	O:35	Alachua	DMSO12	UIA
Serratia marcescens	-	-	13880	ATCC
Shigella flexneri	-	-	12022	ATCC

[1] *Salmonella* Serogroup information was obtained from the WHO Collaborating Centre for Reference and Research on *Salmonella* [1]. For *E. coli*, O-antigen information was provided where available from the indicated source. [2] Strains were obtained from either the American Type Culture Collection (ATCC), the Michigan State University STEC Center (MSU), the University of Georgia (UGA), the Food and Drug Administration (FDA), the United States Department of Agriculture (USDA), or the University of Iowa (UIA).

```
CBA120.NL_TSP3       1 MISQFNQPRGSTSIEVNKQSIARNFGVKEDEVIYFTAGIDLSGFKVIYDESTQRAYSLPFGIVSG  65
RBP-CBA120-1_TSP3    1 MISQFNQPRGSTSIEVNKQSIARNFGVKEDEVIYFTAGIDLSGFKVIYDESTQRAYSLPFGIVSG  65
SPTD1.NL_TSP3        1 MISQFNQPRGSTSIEVNKQSIARNFGVKEDEVIYFTAGIDLSGFKVIYDESTQRAYSLPFGIVSG  65

CBA120.NL_TSP3      66 TTAISLDERAILTHSAGSVDLGELAVSREEYVTLPGSFNFGHTINVKNELLVHDDKKYRWDGSLP 130
RBP-CBA120-1_TSP3   66 TTAISLDERAILTHSAGSVDLGELAVSREEYVTLPGSFNFGHTINVKNELLVHDDKKYRWDGSLP 130
SPTD1.NL_TSP3       66 TTAISLDERAILTHSAGSVDLGELAVSREEYVTLPGSFNFGHTINVKNELIVHDDKRYRWDGSLP 130

CBA120.NL_TSP3     131 KVVAAGSTPDSSGGVGLGAWLSVGDAALRAELNTKVSDGTFPATIKYKYGLPSVIDGAIYRTVQD 195
RBP-CBA120-1_TSP3  131 KVVAAGSTPDSSGGVGLGAWLSVGDAAFRQEANKKFKYSVKLSD----Y---STLQDAATAAVDG 188
SPTD1.NL_TSP3      131 KVVDAGSTPDSSGGVGLGAWLSVGDAAFRQEANKKFKYSVKLSD----Y---STLQDAATAAVDG 188

CBA120.NL_TSP3     196 KLDDFVFL------EDFGGKDDAGSTDNSIAFRKAFA-SGARKIRLRGSGVYGMATRDIELPAKY 253
RBP-CBA120-1_TSP3  189 LLIDINYNFTDGESVDFGGKIL------TINCKAKFIGDGALIFNNMGPGSV------INQP--- 238
SPTD1.NL_TSP3      189 LLIDINYNFTDGESVDFGGKIL------TINCKAKFIGDGALIFNNMGPGSV------INQP--- 238

CBA120.NL_TSP3     254 EIIGNAKNPEI--------KYLGT----DTSFTMFTLTGSGPASNQWKQGGMFRDLIISSDVKIN 306
RBP-CBA120-1_TSP3  239 -FMESKTTPWVIFPWDADGKWITDAALVAATLKQSKIEGYQPGVNDWVKFPGLEALLPQ-NVKDQ 301
SPTD1.NL_TSP3      239 -FMESKTTPWVIFPWDADGKWITDAALVAATLKQSKIEGYQPGVNDWVKFPGLEALLPQ-NVKDQ 301

CBA120.NL_TSP3     307 WMLGRHVQNLDYDRVFFYNSATVLNNYHYVNFTRCERWGSAFIGRADLNTIQFISESP------- 364
RBP-CBA120-1_TSP3  302 HI-TATLDIRSASRVEIRNAGGLMAAYLFRSCHHCKVIDSDSIIGGKDGIITFENLSGDWGLGNY 365
SPTD1.NL_TSP3      302 HI-TATLDIRSASRVEIRNAGGLMAAYLFRSCHHCKVIDSDSIIGGKDGIITFENLSGDWGLGNY 365

CBA120.NL_TSP3     365 -------------------------------------------------------KFHLCFSSG- 373
RBP-CBA120-1_TSP3  366 VIGGRVHYGSGSGVQFLRNNGGESHNGGVIGVTSWRAGESGFKTYQGSVGGGTARNYNLQFRDSV 430
SPTD1.NL_TSP3      366 VIGGRVHYGSGSGVQFLRNNGGESHNGGVIGVTSWRAGESGFKTYQGSVGGGTARNYNLQFRDSV 430

CBA120.NL_TSP3     374 SPIDVWDTADLAITKCTMFAGDYAVRTRVTQKQVTAPDLFAGYPVLITCSVFDAVRGHAWDLEGS 438
RBP-CBA120-1_TSP3  431 ALSPVWDGFDLGSDPGMA----------------PEPDRPGDLPI--SEYPFHQLPNN-----HL 472
SPTD1.NL_TSP3      431 ALSPVWDGFDLGSDPGMA----------------PEPDRPGDLPI--SEYPFHQLPNN-----HL 472

CBA120.NL_TSP3     439 VYSTITGNLVSAGRDTNSHGAYIKGGRSLSLTGNVFTYCGNYGLVLEDVQQSGFVGNVFNGNKTG 503
RBP-CBA120-1_TSP3  473 VDNILVMNSLGVGLGMDGRGGYVSNV--------TVQDCAGAGML----------AHTYNRV-FS 518
SPTD1.NL_TSP3      473 VDNILVMNSLGVGLGMDGRGGYVSNV--------TVQDCAGAGML----------AHTYNRV-FS 518

CBA120.NL_TSP3     504 GLGTLACKDL-------SIVGGSMGTTYVRGGYYTQPVGYSDISSN-S----------TGILLSG 550
RBP-CBA120-1_TSP3  519 NITVIDCNYLNFDSDQIIIIGDCIVNGIKAAGIKPQPSNGLVISAPNSTISGLVGNVPPDKILVG 583
SPTD1.NL_TSP3      519 NITVIDCNYLNFDSDQIIIIGDCIVNGIKAAGIKPQPSNGLVISAPNSTISGLVGNVPPDKILVG 583

CBA120.NL_TSP3     551 VAFDEALTTKVYLDTSITTRNKVINCSGVPDTIARGSTANRPANPQASYQYYDTTLGIPIWWNSV 615
RBP-CBA120-1_TSP3  584 NLLDPVLGQSRVIGFNSDTAELALRINKLSATLDSGALR----------SHLNGSAGSGSAWTEL 638
SPTD1.NL_TSP3      584 NLLDPVLGQSRVIGFNSDTAELALRINKLSATLDSGALR----------SHLNGSAGSGSAWTEL 638

CBA120.NL_TSP3     616 ---SGTWKNAAGADV-------------------------------------------------- 627
RBP-CBA120-1_TSP3  639 TALSGSTPNAVSLKVNRGDYKTTELPISGTVLPDEGVLDINTMSLYLDAGALWALIRLPDGSKTR 703
SPTD1.NL_TSP3      639 TALSGSTPNAVSLKVNRGDYKTTELPISGTVLPDEGVLDINTMSLYLDAGALWALIRLPDGSKTR 703

CBA120.NL_TSP3         -----
RBP-CBA120-1_TSP3  704 MKLSV                                                             708
SPTD1.NL_TSP3      704 MKLSV                                                             708
```

Figure S1. Alignment of TSP3 from CBA120.NL, SPTD1.NL, and RBP-CBA120-1. N-terminal regions native to the recipient, CBA120.NL, are indicated in blue. C-terminal regions derived from the donor, SPTD1.NL, are indicated in red. Alignment was performed by Clustal Omega and visualized with Jalview [2-4].

```
CBA120.NL_TSP2          1 MGYFQMTRNVEELFGGVITAPHQIPFTYKSNVGGETFLSLPFYPVTGVVTINGGMQVPLDNFEIE  65
RBP-CBA120-2_TSP2       1 MGYFQMTRNVEELFGGVITAPHQIPFTYKSNVGGETFLSLPFYPVTGVVTINGGMQVPLDNFEIE  65
SPTD1.NL_TSP2           1 MGYFQMTRNVEELFGGVITAPHQIPFTYKSNVGGETFLSLPFYPVTGVVTINGGMQVPLDNFEIE  65

CBA120.NL_TSP2         66 GNTLNLGRALSKGDVVYCLFDKILSPEDTAKGIRIYKFQAVGGETEFTPDFTSYGVQSLYIGGEY 130
RBP-CBA120-2_TSP2      66 GNTLNLGRALSKGDVVYCLFDKILSPEDTAKGIRIYKFQAVGGETEFTPDFTSYGVQSLYIGGEY 130
SPTD1.NL_TSP2          66 GNMLNLGRALSKGDVVYCLFDKILSPEDTAKGIRIYKFQAVGGETEFTPDFTSYGVQSLYIGGEY 130

CBA120.NL_TSP2        131 KTPEIEYSYDSTTGKVSLQTALSAGVWVVAEMSVKQPNISPAFDRSIQEIARSANVKDSEVIVST 195
RBP-CBA120-2_TSP2     131 KTPEIEYSYDSTTGKVSLQTALSAGVWVVAEMSVKQPNISPAFDRSIQEIARSANVKDSEVIVST 195
SPTD1.NL_TSP2         131 KTPEIEYSYDSTTGKVSLQTALTAGVWVVAEMSVKQPNISPLFDRSIQEIARSANVKDSEVIVST 195

CBA120.NL_TSP2        196 DTISLLDGKKVVYDIATQTSYGLPTIPDGSVISSVSAGKLNYNPGDVQVDLLPLEDSFINVINTL 260
RBP-CBA120-2_TSP2     196 DTISLLDGKKVVYDIATQTSYGLPTIPDGSVISSVSAGKLNYNPGDVQVDLLPLPNSAALLREEL 260
SPTD1.NL_TSP2         196 DTISLLDGKKVVYDIATQTSYGLPTIPDGSVISSVSGGKLNYNPGDVQVNLLPLPNSAALLREEL 260

CBA120.NL_TSP2        261 GRNDGAKYIGECHSVAD--LRNTEPTMDGQRIILKQHTAGTLLGGGVFRALIDGTGKTDNNGTVI 323
RBP-CBA120-2_TSP2     261 GSPTGTNLVNFKNSAVGALVRSVTSKLEDNPISVKDY-------------GCKGDGVTDDTAGLQ 312
SPTD1.NL_TSP2         261 GSPTGTNLVNFKNSAVGALVRSVTSKLEDNPISVKDY-------------GCKGDGVTDDTAGLQ 312

CBA120.NL_TSP2        324 KTVGGAA-WLRVNADRVNPFMFGALGGSNDDTIPVQSCVDSGKATQLTDAHYVSNIQLKYNTSSI 387
RBP-CBA120-2_TSP2     313 LAVAGALSGLVIYFPK--GIYLTSL--PLNFTKPVTLIGEKGARIELTGGDHDYVIQFDFRDTDL 373
SPTD1.NL_TSP2         313 LAVAGALSGLVIYFPK--GIYLTSL--PLNFTKPVTLIGEKGARIELTGGDHDYVIQFDFRDTDL 373

CBA120.NL_TSP2        388 YGSGLHYSRLHQLPSATGNCITIKDTCSLIVLDAFGVYGTGAQQGTSFTAGTTGIYVETPSGLSA 452
RBP-CBA120-2_TSP2     374 MYWGYE------------------AYMSNIIVDG------------------------------- 389
SPTD1.NL_TSP2         374 MYWGYE------------------AYMSNIIVDG------------------------------- 389

CBA120.NL_TSP2        453 DYPFHTTADPRRDLCISKVHIAGFDEYGLNIDSGNFSVTTDSLLVNHINQVGVRCATTDWTWTNI 517
RBP-CBA120-2_TSP2     390 ---------------------KGFAKDGFSLR-GVISSRFDNLRATNISRAGLHCW---WTQCNL 429
SPTD1.NL_TSP2         390 ---------------------KGFAKDGFSLR-GVISSRFDNLRATNISRAGLHCW---WTQCNL 429

CBA120.NL_TSP2        518 QV-NTCGKQCLVLDGCGNGRIIGGKFIWANWQPYGTVGQFPGITINN---SQN---------MVI 569
RBP-CBA120-2_TSP2     430 YNNFHCSKNVELF---------------TTTPKYGILIDYEEGSPNKRGTSADTFINPVCEHTTE 479
SPTD1.NL_TSP2         430 YNNFHCSKNVELF---------------TTTPKYGILIDYEEGSPNKRGTSADTFINPVCEHTTE 479

CBA120.NL_TSP2        570 NGIEVQDCGG------------NGIEISESYSISMNGLNTNRNGINANNTFYNIVFNKSDAVING 622
RBP-CBA120-2_TSP2     480 AGIEATFCINTIFMNGTSEGCRFGIAFGHESDLEQTAVTNTVIGMDMEVNSDGDVLLRKTATSNL 544
SPTD1.NL_TSP2         480 AGIEATFCINTIFMNGTSEGCRFGIAFGHESDLEQTAVTNTVIGMDMEVNSDGDVLLRKTATSNL 544

CBA120.NL_TSP2        623 FVGLNYAANSGS----G------ANSSAGNFQFLSNDCSVTINGVVETGYMGINFIGDNNIINPT 677
RBP-CBA120-2_TSP2     545 FLGVMAGYSSPSSQIQGAEFNRWIGGSSSGFHFYGGTKGNKIEGTM--------LLGASATILDD 601
SPTD1.NL_TSP2         545 FLGVMAGYSSPSSQIQGAEFNRWIGGSSSGFHFYGGTKGNKIEGTM--------LLGASATILDD 601

CBA120.NL_TSP2        678 NSDLSINGLVNYSKT---GL-QTMNETPTFDGVST---TPVYVSVPSSVGQVNGLRLSQANKDKL 735
RBP-CBA120-2_TSP2     602 DKTNDWDSLYNISNATYTGSTNPVNKRVQHDGTGTVTIDPMKCSLA----TINAT-----SSTIT 657
SPTD1.NL_TSP2         602 DKTNDWDSLYNISNATYTGSTNPVNKRVQHDGTGTVTIDPMKCSLA----TINAT-----SSTIT 657

CBA120.NL_TSP2        736 LYSRTAGPEGITMAAVIVPTISGAEVFNFMAIGSGFSDTSNSLHLQLVIDASGKQTIALLLGGDG 800
RBP-CBA120-2_TSP2     658 INTLDNARDGMR--------------------------------MDMIIYNTSGQPLTVNWG--- 687
SPTD1.NL_TSP2         658 INTLDNARDGMR--------------------------------MDMIIYNTSGQPLTVNWG--- 687

CBA120.NL_TSP2        801 TTQILSGDLPNDLKLQSGVPYHIAIGAKPGYFWWSILNIQTGKRIRRSFRGAYLAVPFNSIFGLT 865
RBP-CBA120-2_TSP2     688 DSFRIGGFVPPESGKQKGLSFVW----DANFAHWYCVGMSADGLVY------------------- 729
SPTD1.NL_TSP2         688 DSFRIGGFVPPESGKQKGLSFVW----DANFAHWYCVGMSADGLVY------------------- 729

CBA120.NL_TSP2        866 SSLTFFSDSNAGGDACSGVGAKVYYVGMFSSENDYVASRYYNLINPVDPTKLISYRILDSSI    926
RBP-CBA120-2_TSP2         --------------------------------------------------------------
SPTD1.NL_TSP2             --------------------------------------------------------------
```

Figure S2. Alignment of TSP2 from CBA120.NL, SPTD1.NL, and RBP-CBA120-2. N-terminal regions native to the recipient, CBA120.NL, are indicated in blue. C-terminal regions derived from the donor, SPTD1.NL, are indicated in red. Alignment was performed by Clustal Omega and visualized with Jalview [2-4].

```
SPTD1.NL_TSP4          1 MANKPTQPLFPLGLETSESSNIKGFNNSGTIEHSPGAVMTFPEDTEVTGLPSSVRYNPDSDEFEG   65
RBP-SPTD1-1_TSP4       1 MANKPTQPLFPLGLETSESSNIKGFNNSGTIEHSPGAVMTFPEDTEVTGLPSSVRYNPDSDEFEG   65
CBA120.NL_TSP4         1 MANKPTQPLFPLGLETSESSNIKGFNNSGTIEHSPGAVMTFPEDTEVTGLPSSVRYNPDSDEFEG   65

SPTD1.NL_TSP4         66 YYENGGWLSLGGGGIRWETLPHAPSSNLLEGRGYLINNTTGTSTVVLPSPTRIGDSVTICDAYGK  130
RBP-SPTD1-1_TSP4      66 YYENGGWLSLGGGGIRWETLPHAPSSNLLEGRGYLINNTTGTSTVVLPSPTRIGDSVTICDAYGK  130
CBA120.NL_TSP4        66 YYENGGWLSLGGGGIRWETLPHAPSSNLLEGRGYLINNTTGTSTVVLPSPTRIGDSVTICDAYGK  130

SPTD1.NL_TSP4        131 FATYPLTVSPSGNNLYGSTEDMAITTDNVSATFTWSGPEQGWVITSGVGLGQGRVYSREIFTQIL  195
RBP-SPTD1-1_TSP4     131 FATYPLTVSPSGNNLYGSTEDMAITTDNVSATFTWSGPEQGWVITSGVGLGQGRVYSREIFTQIL  195
CBA120.NL_TSP4       131 FATYPLTVSPSGNNLYGSTEDMAITTDNVSATFTWSGPEQGWVITSGVGLGQGRVYSREIFTQIL  195

SPTD1.NL_TSP4        196 ASETSAVTLNTPPTIVDVYADGKRLAESKYSLDGNVITFSPSLPASTELQVIEYTPIQLGNGGGS  260
RBP-SPTD1-1_TSP4     196 ASETSAVTLNTPPTIVDVYADGKRLAESKYSLDGNVITFSPSLPASTELQVIEYTPIQLGNGGGS  260
CBA120.NL_TSP4       196 ASETSAVTLNTPPTIVDVYADGKRLAESKYSLDGNVITFSPSLPASTELQVIEYTPIQLGNGGGS  260

SPTD1.NL_TSP4        261 GSSTITWVYNGGSAIGGETEITLDVVVDDVPAIDINGSRQYKNLGFTFDPLTSKITLAQELDAED  325
RBP-SPTD1-1_TSP4     261 GSSTITWVYNGGSAIGGETEITLDVVVDDVPAIDINGSRQYKNLGFTFDPLTSKITLAQELDAED  325
CBA120.NL_TSP4       261 GSSTITWVYNGGSAIGGETEITLDIVVDDVPAIDINGSRQYKNLGFTFDPLTSKITLAQELDAED  325

SPTD1.NL_TSP4        326 EVVVIINGTPNIYNQIDYTLREVARVTNVKDTEVVYFSVGAVLSGYKVIYDKVTQRSYFIPELPT  390
RBP-SPTD1-1_TSP4     326 EVVVIINGTPNIYNQIDYTLREVARVTNVKDTEVVYFSVGAVLSGYKVIYDKVTQRSYFIPELPT  390
CBA120.NL_TSP4       326 EVVVIINGTPNIYNQIDYTLREVARVTNVKDTEVIYFSVGAVLSGYKVIYDKVTQRSYFIPELPT  390

SPTD1.NL_TSP4        391 GTTAVSLSSSAILVHSAGSVDLGALAVSREEYVTLSGTFDSGAVINTKNELLTHTDGKYRWDGAL  455
RBP-SPTD1-1_TSP4     391 GTTAVSLSSSAILVHSAGSVDLGALAVSREEYVTLSGTFDSGAVINTKNELLTHTDGKYRWDGAL  455
CBA120.NL_TSP4       391 GTTAVSLSSSAILVHSAGSVDLGALAVSREEYVTLSGTFDSGAVINTKNELLTHTDGKYRWDGTL  455

SPTD1.NL_TSP4        456 PKTVDAGSTPETSGGIGIGSWVGVGDASLRSDLNSTNGAGIVKTGNGNTVQETFDFIKNV-QQTN  519
RBP-SPTD1-1_TSP4     456 PKTVDAGSTPETSGGIGIGSWVGVGDASLKSNLNKPNGLSYIGTVSSV--SELSSIAGLIGDSII  518
CBA120.NL_TSP4       456 PKTVAAGSTPATTGGVGSGAWLSVGDASLKSNLNKPNGLSYIGTVSSV--SELSSIAGLIGDSII  518

SPTD1.NL_TSP4        520 LHPFVPSGNFGSGLPFDSSLQVFKYNDEYYSYKGPIVPGSIVPATPSVDSNWKKEPVSDYIENYG  584
RBP-SPTD1-1_TSP4     519 LDSYVDGFNLGGGVMVAVNSDTV--VDNIVTF-------------QGNGVVWKRKLFNGVADVYE  568
CBA120.NL_TSP4       519 LDSYVDGFNLGGGVMVAVNSDTV--VDNIVTF-------------QGNGVVWKRKLFNGVADVYE  568

SPTD1.NL_TSP4        585 GGVHVADQTPAIMAAIS-AGVRRIIFPKGE--------------LRMSQCDIP------------  622
RBP-SPTD1-1_TSP4     569 AGYTGTGDLAIFINKINAVGFDCIVPVSGEITTPIIFDIAKGALIGKNKCTLIESASATGDYYLT  633
CBA120.NL_TSP4       569 AGYTGTGDLAIFINKINAVGFDCIVPVSGEITTPIIFDIAKGALIGKNKCTLIESASATGDYYLT  633

SPTD1.NL_TSP4        623 -----------------EYTQGLTFEGQGANQAYTGTTVIKPLN--DNQNCLFNSGVNQH-----  663
RBP-SPTD1-1_TSP4     634 IVNTDTDYTNRDVINATALMTGVSFVGKGTRKLAIGGSTSGEVSELRISNCGFISTAGIEFLDNA  698
CBA120.NL_TSP4       634 IVNTDTDYTNRDVINATALMTGVSFVGKGTRKLAIGGSTSGEVSELRISNCGFISTAGIEFLDNA  698

SPTD1.NL_TSP4        664 -------------GQDSIKF-------KNIQFDGEWRCNHAILHESGAGWYYEKLTGKNFNTWVI  708
RBP-SPTD1-1_TSP4     699 YRILFDKCALSRSFTNSVIFNSPANSGEVIKFNHCWMVD------NGGPFTFKN-------GQFI  750
CBA120.NL_TSP4       699 YRILFDKCALSRSFTNSVIFNSPANSGEVIKFNHCWMVD------NGGPFTFKN-------GQFI  750

SPTD1.NL_TSP4        709 YDDQGLTVMRDVYTEATSSDLTKRATGGCIRIYSDSVMDTVNCYGGSVPLWIAAGGNRITNIWAN  773
RBP-SPTD1-1_TSP4     751 FDSCSLP---------------AGKKSG----YFDPVVA---------------LSDNATTVFTN  781
CBA120.NL_TSP4       751 FDSCSLP---------------AGKKSG----YFDPVVA---------------LSDNATTVFTN  781

SPTD1.NL_TSP4        774 GGYDVGAIRLMPKNDSTNHINTAMSNLYIGESTNE---SGTAIPVLELQGNSIRRVSGVQISNAH  835
RBP-SPTD1-1_TSP4     782 GNI-----EYQPGQSFVGFTVDGSSRLSISDSTILLPNDYSTVPIVN-NGDGVVSL-----NN--  833
CBA120.NL_TSP4       782 GNI-----EYQPGQSFVGFTVDGSSRLSISDSTILLPNDYSTVPIVN-NGDGVVSL-----NN--  833

SPTD1.NL_TSP4        836 FVHADLTLNTNMVMIRTDKVDGFVM------SSFDVLGRGEYSSASSRTYNFLNATD----STGM  890
RBP-SPTD1-1_TSP4     834 ---CSLPLYG-----STTIATGFATRQLIGGLSKKIMSRGCYPRAGFITSNWNLGCIVSPYINSV  890
CBA120.NL_TSP4       834 ---CSLPLYG-----STTIATGFATRQLIGGLSKKIMSRGCYPRAGFITSNWNLGCIVSPYINSV  890

SPTD1.NL_TSP4        891 AFSAGSVRGINN--------------------NPIVLSSGSRLAI-------------------D  916
RBP-SPTD1-1_TSP4     891 SNGSGQFENISNWTLSQTGTDVVTVTTGNDVPNDLMFSTSFVLSVPTVGAAANFTQTIIDCEPGR  955
CBA120.NL_TSP4       891 SNGSGQFENISNWTLSQTGTDVVTVTTGNDVPNDLMFSTSFVLSVPTVGAAANFTQTIIDCEPGR  955

SPTD1.NL_TSP4        917 AVEFDGWSTNSDSGIENKAAIRSSGAGTRCCIGSSCAFNASQADAYAVYAGDEFAV-DISILNLN  980
RBP-SPTD1-1_TSP4     956 YFQLGFWAKNTTTTLASIRFLDQQGNAVAD----SIGYNIPVGNTFNFYALVDCVPPGAYKAEIN 1016
CBA120.NL_TSP4       956 YFQLGFWAKNTTTTLASIRFLDQQGNAVAD----SIGYNIPVGNTFNFYALVDCVPPGAYKAEIN 1016

SPTD1.NL_TSP4        981 YPSSNII---IPANTNYSYMYKRPGGKLTLVNTLTI                             1013
RBP-SPTD1-1_TSP4    1017 FNVSSIVGGIAIHNVIYGLI----------------                             1036
CBA120.NL_TSP4      1017 FNVSSIVGGIAIHNVIYGLI----------------                             1036
```

Figure S3. Alignment of TSP4 from CBA120.NL, SPTD1.NL, and RBP-SPTD1-1. N-terminal regions native to the recipient, SPTD1.NL, are indicated in blue. C-terminal regions derived from the donor, CBA120.NL, are indicated in red. Alignment was performed by Clustal Omega and visualized with Jalview [2-4].

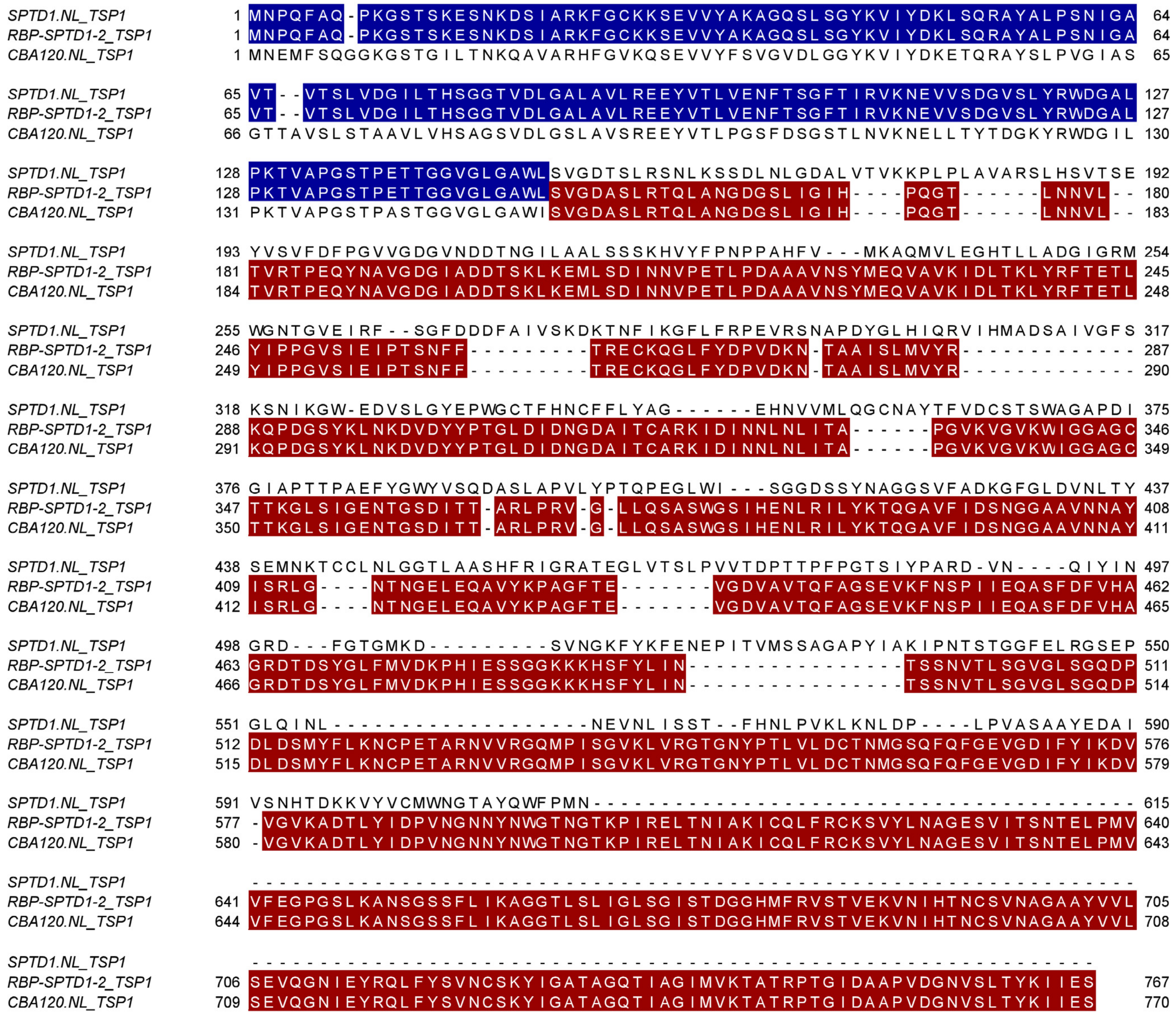

Figure S4. Alignment of TSP1 from CBA120.NL, SPTD1.NL, and RBP-SPTD1-2. N-terminal regions native to the recipient, SPTD1.NL, are indicated in blue. C-terminal regions derived from the donor, CBA120.NL, are indicated in red. Alignment was performed by Clustal Omega and visualized with Jalview [2-4].

```
RBP-SPTD1-2_TSP2    1 MGYFQMTRNVEELFGGVITAPHQIPFTYKSNVGGETFLSLPFYPVTGVVTINGGMQVPLDNFEIE  65
RBP-SPTD1-3_TSP2    1 MGYFQMTRNVEELFGGVITAPHQIPFTYKSNVGGETFLSLPFYPVTGVVTINGGMQVPLDNFEIE  65
Det7_TSP2           1 MGYLQMTRNVESIFGAVVTAPHQIPYTYTA-TGGETFISLPFYPVTGFITINGGVQVPVDNYEID  64

RBP-SPTD1-2_TSP2   66 GNMLNLGRALSKGDVVYCLFDKILSPEDTAKGIRIYKFQAVGGETEFTPDFTSYGVQSLYIGGEY 130
RBP-SPTD1-3_TSP2   66 GNMLNLGRALSKGDVVYCLFDKILSPEDTAKGIRIYKFQAVGGETEFTPDFTSYGVQSLYIGGEY 130
Det7_TSP2          65 GNTVNLGRALEADDVVYCLFDKILSPEDYENGIRIYKFQAVGNETTFTPDFTTYGVQTLYIDGKF 129

RBP-SPTD1-2_TSP2  131 KTPEIEYSYDSTTGKVSLQ-TALTAGVWVVAEMSVKQPNISPLFDRSIQEIARSANVKDSEVIVS 194
RBP-SPTD1-3_TSP2  131 KTPEIEYSYDSTTGKVSLQ-TALTAGVWVVAEMSVKQPNISPLFDRSIQEIARSANVKDSEVIVS 194
Det7_TSP2         130 QVPGVDYNYNSATGVVSFLNGSPTAGVWVVAEMSIKQNYLALSSDSGASLVGTSSGNTVQEVLNS 194

RBP-SPTD1-2_TSP2  195 T-----DTISLLDGKKVVYDIA--TQTSYGLPTIPDGSVISSVSGGKLNYNPGDVQVNLLPLPNS 252
RBP-SPTD1-3_TSP2  195 T-----DTISLLDGKKVVYDIA--TQTSYGLPTIPDGSVISSVSGGKLNYNPGDVQVNLLPLPNS 252
Det7_TSP2         195 HSGSFQTGVKLLSSNDLIVDQSVIPNQLYRW----DGAFPKTVAAGSSPASTGGVGN-GSWVSVG 254

RBP-SPTD1-2_TSP2  253 AALLREELGSPTGTNLV---NFKNSAVGALVRSVTSKLEDNPISVKDYGCKGDGVTDDTAGLQLA 314
RBP-SPTD1-3_TSP2  253 AALLRGELNNEGVINFSHADTYGNDSVGAHLQNVVYPT-DA-----PFNAATDGTTDTTVAIKSA 311
Det7_TSP2         255 DATLRGELNNEGVINFSHADTYGNDSVGAHLQNVVYPT-DA-----PFNAATDGTTDTTVAIKSA 313

RBP-SPTD1-2_TSP2  315 VAGALSGLVIYFPKGIYLTSLPLNFTKP---VTLIGEKGARIELTGGDHDYVIQ----------- 365
RBP-SPTD1-3_TSP2  312 IAHCISKGKKLVLNHLFMITDTLVISDGLHVECLTSDSGVKSDVPAGKFAVKITGANSGWFGGKI 376
Det7_TSP2         314 IAHCISKGKKLVLNHLFMITDTLVISDGLHVECLTSDSGVKSDVPAGKFAVKITGANSGWFGGKI 378

RBP-SPTD1-2_TSP2  366 --------FDFRDTDLMYWGYEAYMSNIIVDGKGFAKDGFSLRGVISSRFDNLRATNISRAGLHC 422
RBP-SPTD1-3_TSP2  377 LGKNLPESTTVRQDGVLFDENAEYCFI-----TGTEVTGFFAKGLHTSDADGVGY-GIYDKGYGT 435
Det7_TSP2         379 LGKNLPESTTVRQDGVLFDENAEYCFI-----TGTEVTGFFAKGLHTSDADGVGY-GIYDKGYGT 437

RBP-SPTD1-2_TSP2  423 WWTQCNLYNNFHCSKNVELFTTTPKYGILIDYEEGSPNKRGTSADTFINPVCEHTTEAGIEATFC 487
RBP-SPTD1-3_TSP2  436 LISKCYANSKFC---------------VALGGTEGRV---------LKNRITNNYLTSGEAKPWS 476
Det7_TSP2         438 LISKCYANSKFC---------------VALGGTEGRV---------LKNRITNNYLTSGEAKPWS 478

RBP-SPTD1-2_TSP2  488 INTIFMNGT--SEGCRFGIAFGHESDLEQTAVTNTVIGMDMEVNSDGDVLLRKTATSNLFLGVMA 550
RBP-SPTD1-3_TSP2  477 WASNYWDGIVSENAHRYVIAFNDVSA---------------------------CGQSGIYFGGNG 514
Det7_TSP2         479 WASNYWDGIVSENAHRYVIAFNDVSA---------------------------CGQSGIYFGGNG 516

RBP-SPTD1-2_TSP2  551 GYSSPSSQIQGAEFNRWIGGSSSGFHFYGGTKGNKI-------------EGTM-LLGASATILDD 601
RBP-SPTD1-3_TSP2  515 GYSTDNIIVNNTVYACWNRGIDMGLFSEKSATNDVLRNIIKGNNTYNNRENNIWLAGVSN-CSVV 578
Det7_TSP2         517 GYSTDNIIVNNTVYACWNRGIDMGLFSEKSATNDVLRNIIKGNNTYNNRENNIWLAGVSN-CSVV 580

RBP-SPTD1-2_TSP2  602 DKTNDWDSLYNISNATYTGSTNPVNKRVQHDGTG--TVTIDPMKCSLATIN-------------- 650
RBP-SPTD1-3_TSP2  579 GNTSWFDTNYDVIFAGYPGGHICISLASGANGEACVGNTIDSNTCIDPRGNAGITVPTGATGNVF 643
Det7_TSP2         581 GNTSWFDTNYDVIFAGYPGGHICISLASGANGEACVGNTIDSNTCIDPRGNAGITVPTGATGNVF 645

RBP-SPTD1-2_TSP2  651 --------------ATSSTITINTLDNARDGMRMDMIIYNTSGQPLTVNWGDSFR---------- 691
RBP-SPTD1-3_TSP2  644 GSGNNLSQAGAIYIASPDLITSNRFELAVTGSFTPVLLPESGSITLSSSSTGVFRATGNRIDFSV 708
Det7_TSP2         646 GSGNNLSQAGAIYIASPDLITSNRFELAVTGSFTPVLLPESGSITLSSSSTGVFRATGNRIDFSV 710

RBP-SPTD1-2_TSP2  692 --------------IGGFVPPESGKQKGLSFVWDANFAHWYCVGMSADG---------------- 726
RBP-SPTD1-3_TSP2  709 TVNVSSISSPSGNLNIAYLPGMSGKTSSTSMFI---IDYWNDLTLSSGVIPLASLNLENQDQITV 770
Det7_TSP2         711 TVNVSSISSPSGNLNIAYLPGMSGKTSSTSMFI---IDYWNDLTLSSGVIPLASLNLENQDQITV 772

RBP-SPTD1-2_TSP2  727 -------LVY---------------------                                   729
RBP-SPTD1-3_TSP2  771 YRTDGGRVLYDFSSLMKSTSSFILKGFVDFN                                   801
Det7_TSP2         773 YRTDGGRVLYDFSSLMKSTSSFILKGFVDFN                                   803
```

Figure S5. Alignment of TSP2 from Det7, RBP-SPTD1-2, and RBP-SPTD1-3. N-terminal regions native to the recipient, RBP-SPTD1-2, are indicated in blue. C-terminal regions derived from the donor, Det7, are indicated in red. Alignment was performed by Clustal Omega and visualized with Jalview [2-4].

Table S2. Individual Relative Light Unit Values for (Figure 5)

Reporter[1]	Strain[2]	CFU[3]	Well 1[4]	Well 2[4]	Well 3[4]	Well 4[4]	Well 5[4]	Well 6[4]	Well 7[4]	Well 8[4]	Well 9[4]	Well 10[4]
SPTD1.NL	*S.* Typhimurium	0	115	127	135	88	93	94	-	-	-	-
		10	1359	180	516	321	2781	1890	1345	654	2040	3044
		100	11,620	16,550	8250	12,735	18,975	13,450	21,570	20,415	18,485	25,715
		1000	243,450	244,250	233,750	213,750	263,250	284,650	-	-	-	-
		10,000	8,312,500	7,804,500	8,312,000	8,365,500	8,859,000	9,113,500	-	-	-	-
	S. Minnesota	0	87	105	91	98	99	104	-	-	-	-
		10	75	103	105	114	79	92	80	85	76	92
		100	90	98	108	104	94	87	87	87	82	89
		1000	93	103	98	101	84	87	-	-	-	-
		10,000	94	86	103	86	79	81	-	-	-	-
	S. Anatum	0	114	111	100	108	88	109	-	-	-	-
		10	108	84	105	101	106	97	102	110	189	94
		100	97	101	99	103	105	92	96	102	94	108
		1000	98	104	93	94	80	94	-	-	-	-
		10,000	95	94	103	102	89	91	-	-	-	-
	C. sedlakii	0	66	65	86	82	72	76	-	-	-	-
		10	64	72	108	67	65	199	838	112	171	286
		100	1432	1699	2731	776	1139	2450	1778	2768	1401	1829
		1000	14,461	17,057	15,240	16,901	13,564	21,645	-	-	-	-
		10,000	195,074	224,119	191,759	236,887	195,079	253,215	-	-	-	-
RBP-SPTD1-3	*S.* Typhimurium	0	188	171	171	171	164	190	-	-	-	-
		10	2751	2474	2852	2531	5239	1027	1647	5573	3043	4041
		100	18,730	11,325	24,155	16,890	21,175	15,490	24,820	17,550	18,805	28,450
		1000	413,350	343,650	394,700	422,750	361,200	412,600	-	-	-	-
		10,000	11,740,000	11,110,000	11,390,000	11,950,000	12,240,000	11,505,000	-	-	-	-
	S. Minnesota	0	145	162	144	142	166	135	-	-	-	-
		10	263	1924	639	875	799	149	155	218	1637	608
		100	4348	4250	5136	5136	7093	4670	2608	7782	6039	4140
		1000	35,223	87,005	65,298	75,211	62,893	54,851	-	-	-	-
		10,000	718,963	1,267,003	947,478	948,365	1,063,823	1,164,381	-	-	-	-
	S. Anatum	0	171	174	181	162	176	177	-	-	-	-
		10	5397	4947	1846	908	308	4353	4530	3380	1782	4349
		100	54,478	19,589	47,162	20,813	33,370	18,810	56,591	31,789	32,079	17,958
		1000	434,691	273,746	403,911	396,008	399,783	358,322	-	-	-	-
		10,000	10,480,007	7,037,041	10,563,236	9,028,407	9,458,159	9,953,473	-	-	-	-
	C. sedlakii	0	158	141	154	152	156	144	-	-	-	-
		10	144	139	148	155	134	156	138	133	136	126
		100	145	123	133	146	146	160	151	144	149	159
		1000	130	152	141	159	133	147	-	-	-	-
		10,000	136	155	152	143	128	141	-	-	-	-

[1] Working stocks (10 µL of 1.2 × 10^7 PFU per mL) of SPTD1.NL or RBP-SPTD1-3 were used as diagnostic reporters with a two-hour infection. [2] Log-phase cultures of each bacterial strain were prepared from diluted overnight cultures. Strain information is provided elsewhere (Table S1). The *S.* Anatum strain used was SLR 377. [3] Cultures were diluted to the indicated number of colony forming units (CFU) per well. [4] Six to ten replicate wells were prepared for each condition. After infection, Nanoluc® production was assessed using a GloMax® Navigator. Relative light units (RLU) for each well were obtained by averaging two back-to-back readings.

Supplementary Material References

1.Grimont, P.A.D.; Weill, F.-X. *Antigenic Formulae of the Salmonella Serovars*, 9th ed.; WHO Collaborating Centre for Reference and Research on Salmonella; Pasteur Institute: Paris, France, 2007.

2.Rice, P.; Longden, I.; Bleasby, A. EMBOSS: the European Molecular Biology Open Software Suite. *Trends Genet* **2000**, *16*, 276-277, doi:10.1016/s0168-9525(00)02024-2.

3.Sievers, F.; Wilm, A.; Dineen, D.; Gibson, T.J.; Karplus, K.; Li, W.; Lopez, R.; McWilliam, H.; Remmert, M.; Soding, J., et al. Fast, scalable generation of high-quality protein multiple sequence alignments using Clustal Omega. *Mol Syst Biol* **2011**, *7*, 539, doi:10.1038/msb.2011.75.

4.Waterhouse, A.M.; Procter, J.B.; Martin, D.M.; Clamp, M.; Barton, G.J. Jalview Version 2--a multiple sequence alignment editor and analysis workbench. *Bioinformatics* **2009**, *25*, 1189-1191, doi:10.1093/bioinformatics/btp033.
