# Supplementary figures and images for: Tailoring the Host Range of Ackermannviridae Bacteriophages through Chimeric Tailspike Proteins
Source: Viruses. 2023 Jan 19;15(2):286. doi: 10.3390/v15020286 (PMC9965104; doi:10.3390/v15020286)

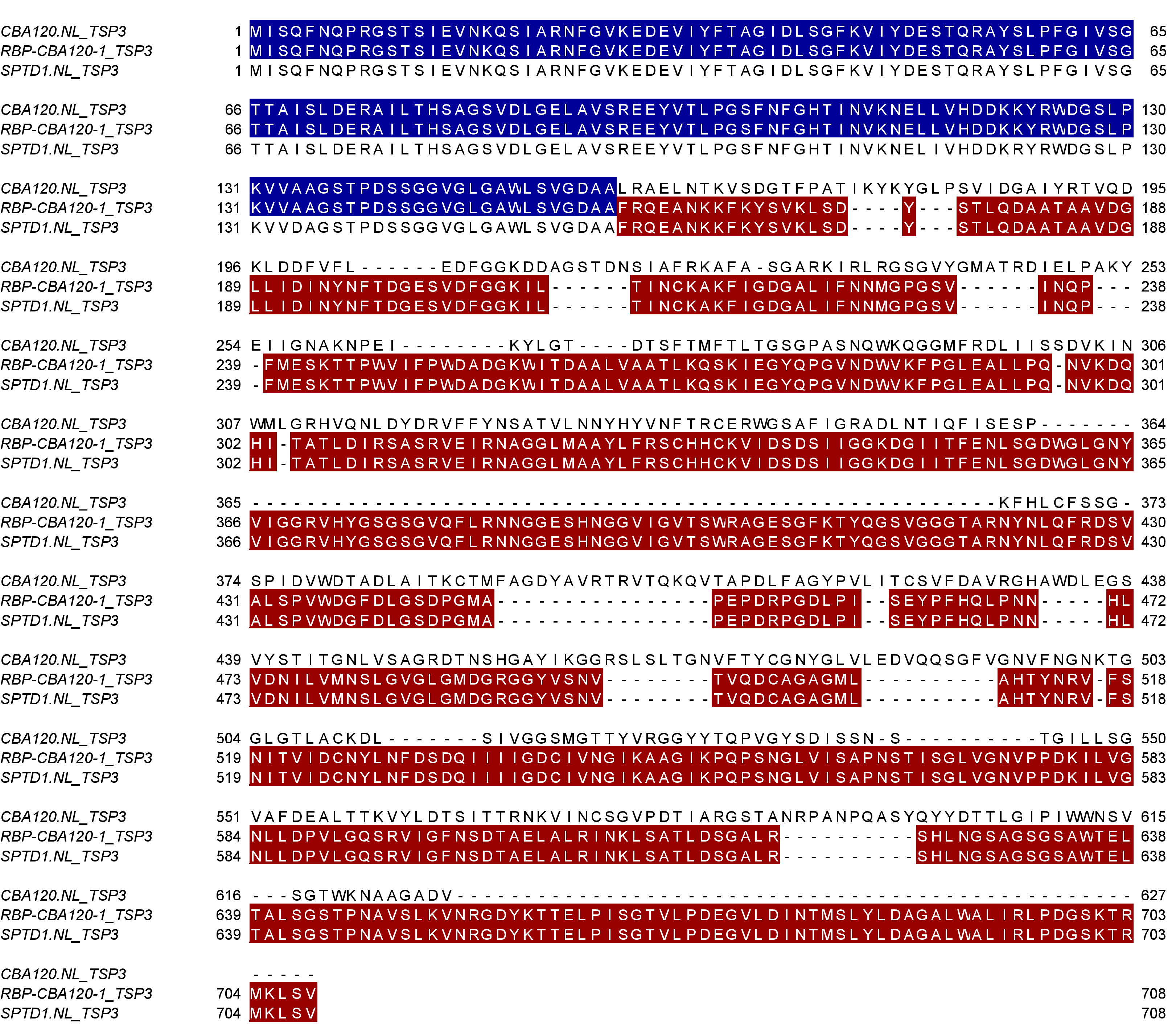

Supplement: Supplementary file 1 [file viruses-15-00286-s001.zip › Figure S1.tif]

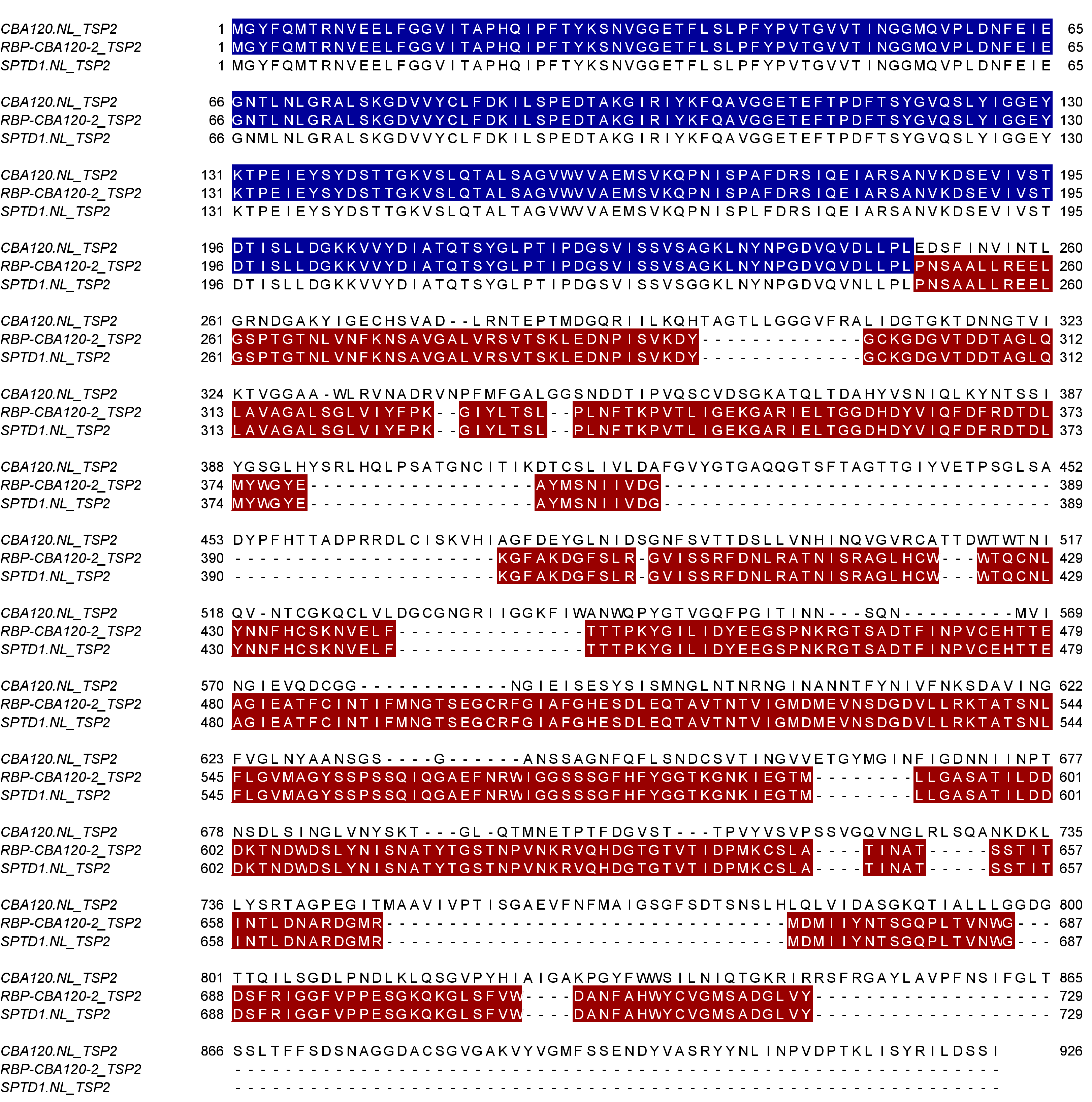

Supplement: Supplementary file 1 [file viruses-15-00286-s001.zip › Figure S2.tif]

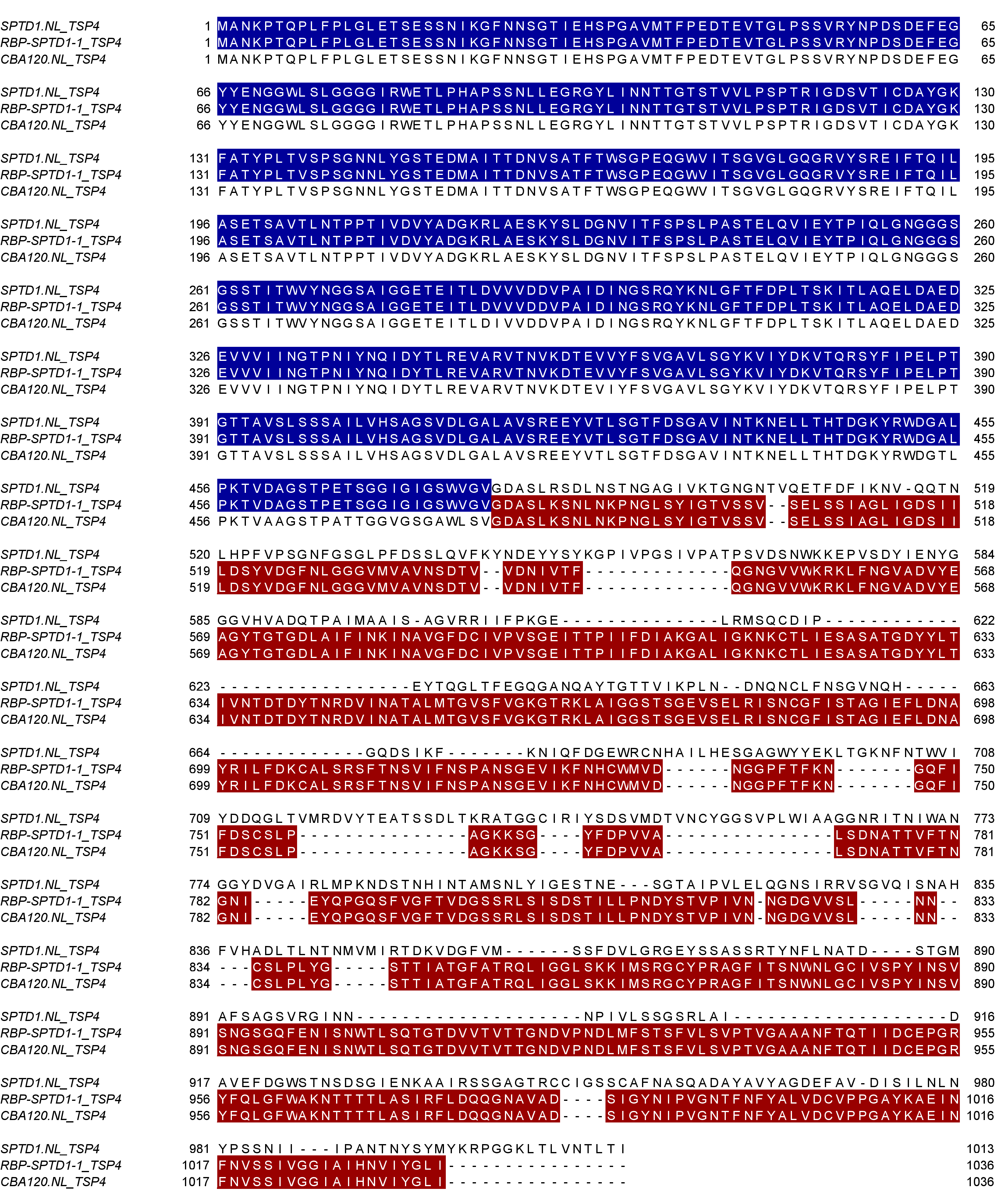

Supplement: Supplementary file 1 [file viruses-15-00286-s001.zip › Figure S3.tif]

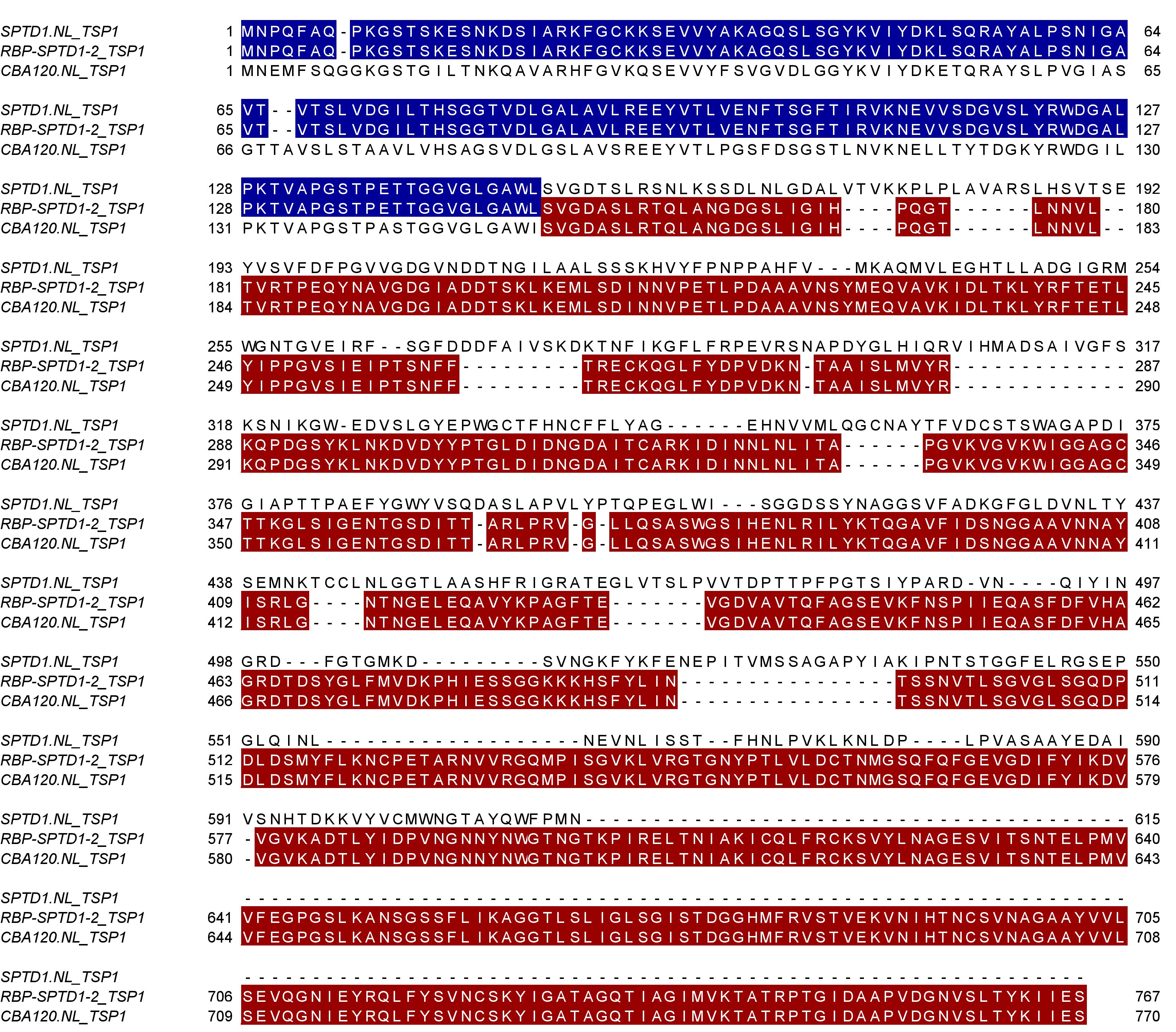

Supplement: Supplementary file 1 [file viruses-15-00286-s001.zip › Figure S4.tif]

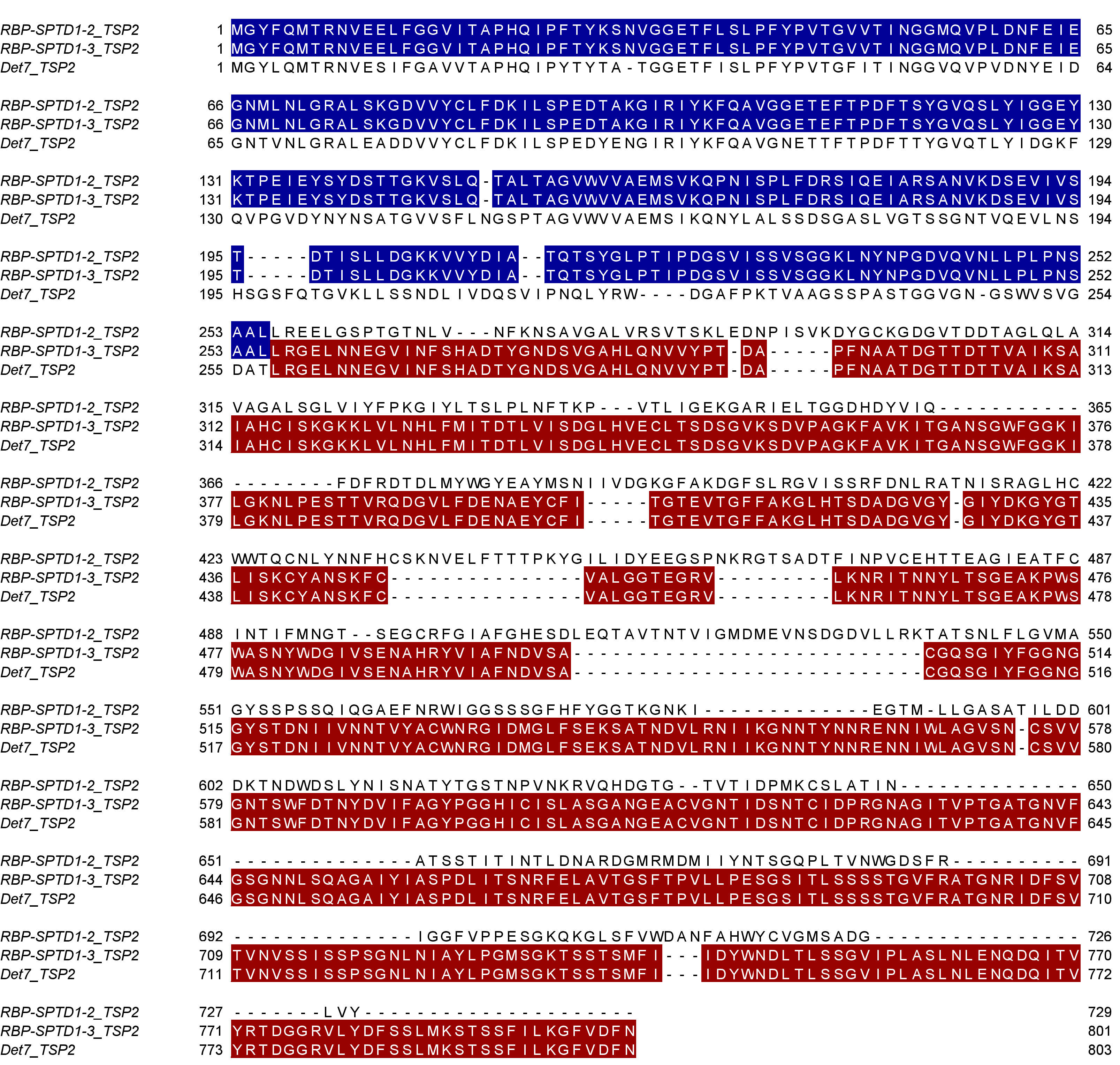

Supplement: Supplementary file 1 [file viruses-15-00286-s001.zip › Figure S5.tif]
